# Oral Hygiene Behavior Among Asylum Seekers and Refugees Using Health Beliefs Model: A Cross-Sectional Study

**DOI:** 10.3389/ijph.2026.1609334

**Published:** 2026-02-23

**Authors:** Lujain Alchalabi, Emmanuel Schaffner, Yaman Maani-Abuzahra, Julia Dötzer, Ayoung Jeong, Nicola U. Zitzmann, Sonja Merten, Gianfranco Lovison, Nicole Probst-Hensch

**Affiliations:** 1 Swiss Tropical and Public Health Institute (Swiss TPH), Allschwil, Switzerland; 2 Universitat Basel, Basel, Switzerland; 3 Department of Reconstructive Dentistry, Universitares Zentrum fur Zahnmedizin Basel, Basel, Switzerland

**Keywords:** dental floss, health belief model, oral hygiene, refugees, toothbrushing

## Abstract

**Objectives:**

This study examined oral hygiene behavior (OHB) and Health Belief Model-based (HBM) determinants among asylum seekers and refugees (ASR).

**Methods:**

Cross-sectional survey data from 300 Arabic-speaking ASRs in Switzerland were analyzed for associations of two OHB outcomes (Adequate: toothbrushing twice a day; interdental cleaning at least each other day) with HBM-based explanatory variables. Mixed logistic regression models were used, adjusting for age, sex, education, and household.

**Results:**

Inadequate-OHB was common (toothbrushing: 47%; interdental cleaning: 65%). ASRs reporting higher self-efficacy under stress, more likely reported adequate-OHB (toothbrushing: odds ratio (95% confidence interval) 2.93 (0.68,12.70); interdental cleaning: 3.10 (2.28,4.22). Barriers (anticipating pain or breakage, lack of time, limited knowledge) were associated with reduced likelihood of adequate-OHB (interdental cleaning: don’t know how: 0.76 (0.61, 0.95)). Benefits were associated with adequate interdental cleaning (making mouth feel better: 1.61 (1.14, 2.27); saving money later: 1.36 (1.01; 1.82)). The likelihood of adequate toothbrushing increased with autonomy (control of decisions on one’s dental health: 1.40 (0.91, 2.17)).

**Conclusion:**

Self-efficacy, autonomy, barriers and benefits may be key OHB determinants among ASRs, but need testing in intervention studies.

## Introduction

Oral health (OH) diseases have a lifelong impact and can lead to reduced quality of life, pain, impaired nutrition, infections, as well as cardio-vascular and other non-communicable diseases [1, 2]. Their global burden is growing [3, 4]. Sustainable daily oral hygiene behavior (OHB) is crucial for preventing common oral diseases such as caries and periodontal disease [5, 6].

According to the World Health Organization, OHB and preventive OH measures have the lowest cost, but the highest demand level in the primary OH care pyramid [4]. The main objective of OHB is to minimize bacterial buildup within dental biofilms on teeth surfaces. Although toothbrushing is the most common method for addressing this, it often fails to effectively clean predilection sites like the interproximal spaces. Therefore, interdental cleaning plays a crucial role in preventing interproximal caries and periodontal disease [7, 8].

Enhancing OHB in an effective and targeted manner depends on the understanding of the key behavioral determinants of OHB. It requires a conceptual framework that accounts for both individual beliefs and contextual factors [9]. The Health Belief Model (HBM) is one of the most widely used theoretical frameworks in health behavior research, offering a useful lens for examining health-related decision-making. According to the HBM, individuals are more likely to engage in health-promoting behaviors when they perceive themselves as susceptible to a condition, believe the condition has serious consequences, recognize the benefits of preventive action, perceive few barriers to taking action, have confidence in their ability to perform the behavior (self-efficacy), and encounter cues to action [10–12]. HBM has been widely applied in OH research, including studies on OHB [13–17].

Enhancing OHB might alleviate the impact of socioeconomic disadvantage in OH [18]. Accordingly, a critical question is how to effectively promote OHB among the population’s subgroups. Displaced populations are of particular interest with regards to preventive health practices. They often have disrupted living environments, competing priorities, and barriers that complicate the adoption and maintenance of such practices. Asylum seekers and refugees (ASR) often face challenges that hinder their ability to conduct consistent OHB, including migration-related stressors before, during and post-migration, limited access to OH care, and financial difficulties [4, 19–22]. Limited contact with OH care services often results in reduced exposure to OH promotion, education, and follow-up, which may further hinder the development of regular and efficient OHB [23]. In addition, many ASRs may arrive with limited OH knowledge due to overstretched healthcare systems in their countries of origin or prolonged periods of instability and movement through camps and temporary settings [24]. Furthermore, OHB is developed in early stages of human’s life and it is impacted by the parents [25], an opportunity potentially unavailable to children of displaced families.

Evidence from previous cross-sectional studies shows a high prevalence of oral diseases among refugees. In Norway, refugees from the Middle East and Africa exhibited a substantial caries burden, with a mean of 4.3 decayed teeth per person [26]. In Germany, 79% showed plaque and 30% showed calculus in all mouth sextants, indicating poor oral hygiene. 80% believed in the relevance of toothbrushing twice a day, whereas 69% reported that flossing is not needed [27]. Among school-age refugee children in Sweden, 48% had untreated caries [28].

The ASR population continues to grow due to ongoing global conflicts and migration trends [29]. This is also true for Switzerland, where OH care isn't covered by the basic insurance and private coverage is not at low costs, making it hard to afford for low-income individuals including ASRs [30, 31]. According to the treatment recommendations, annual dental check-ups and professional oral hygiene must be covered by the state of residence for ASR. Some research has examined the general health needs of ASR populations in Switzerland [9], but not their OH needs. More generally, studies specifically dedicated to determinants of ASRs OHB remain scarce.

### Objectives

**FIGURE 1:**
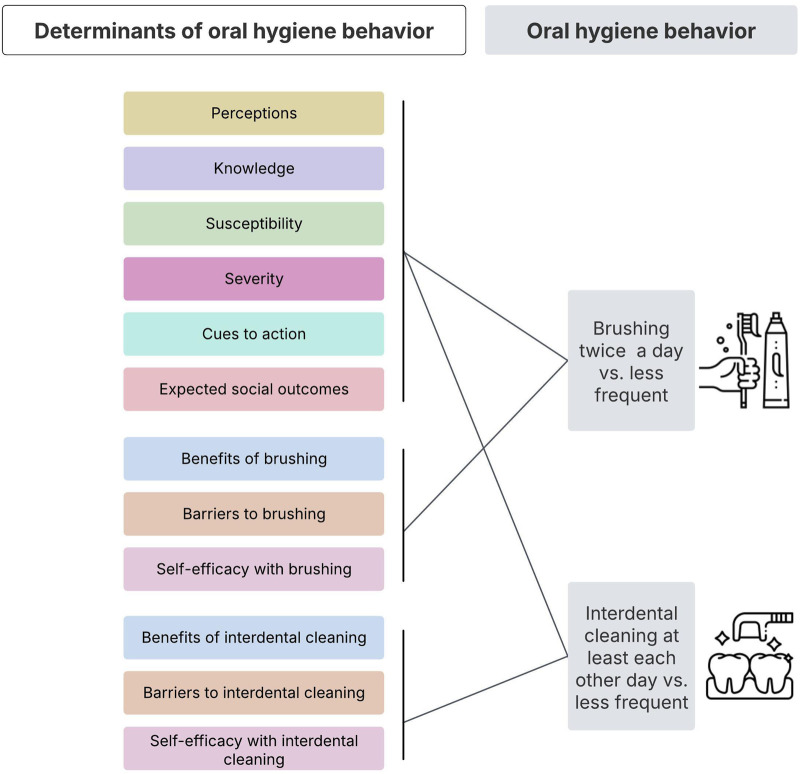
A conceptual Overview of Health Belief Model-based determinants of oral hygiene behavior. (Access to oral healthcare among asylum seekers and refugees, Switzerland, 2025).

The current study aims at exploring OHB (toothbrushing; interdental cleaning) and HBM-based determinants (Figure 1) among adult and adolescent ASRs in North-Western Switzerland. The goal of the current observational study is to identify which specific HBM-based determinants may hold the greatest practical relevance for promoting OHBs, and thus need further more rigorous testing in intervention studies towards a better understanding of causality. Ultimately, the insight gained from the current observational and future intervention studies might guide dental professionals in asking focused questions to better support OHB among ASRs.

## Methods

The research project Axs2OH (access to oral healthcare among asylum seekers and refugees in Switzerland) received approval from the ethics committee of the canton of northwestern Switzerland (EKNZ, No. 2023-00871). It was carried out in line with the revised declaration of Helsinki [32], with written informed consent obtained from all participants beforehand. Data were reported following the STROBE guidelines [33] (Supplementary Table S4).

### Study Design and Population

Eligible participants were: Adolescents aged between 14 and 17 years old, or adults aged 18 and older, who speak Arabic, have permission to stay in Switzerland as ASRs, and live in the states of Zürich, Basel-Stadt, or Basel-Landschaft.

Power calculations were based on 4,634 eligible participants in Zürich, Basel-Stadt, or Basel-Landschaft [34, 35], assuming a 30% response rate. A sample of 284 randomly selected participants was deemed sufficient, accounting for household clustering. This sample size allows detection of an odds ratio (OR) of 2.36 between HBM domains and OHB, with 95% confidence and 80% power.

In the absence of access to a database allowing for population-based recruitment, participants were initially recruited by distributing study flyers in integration centers and dental clinics and thereafter also by snowballing recruitment between September 2024 and March 2025. Interested individuals contacted the study staff by phone for a brief eligibility screening. Appointments were then arranged for eligible and interested participants. They received the informed consent form and a printed and verbal explanation of the study in-person. Following written informed consent, trained study personnel conducted face-to-face, verbally administered surveys with participants using visuals for each response option to aid understanding and accuracy. Responses were securely recorded and stored using the Open Data Kit platform. Questions were asked in Arabic language. Questions were independently translated from English to Arabic and back translated to English by two Arabic speaking members of study staff fluent in English. Available resources did not allow validating the questionnaire, albeit most questions were derived from previously validated and published questionnaires. The comprehensibility of the questions was pilot-tested.

### Variables and Measures

Questions from which outcomes, predictors and covariates were derived are available as Supplementary Material.

Primary outcomes: OHB (oral hygiene behavior), previously defined, was assessed via two self-reported items:

- Adequate toothbrushing (toothbrushing at least twice a day versus less frequent)
- Adequate interdental cleaning (cleaning at least every second day versus less frequent)

This cut-off of adequate interdental cleaning is not a gold-standard recommendation. It was informed by previous research suggesting that interdental cleaning several times a week may offer protective benefits, even if not performed daily [36], and by the distribution of responses in our dataset.

Primary explanatory variables: HBM-based determinants [10] of OHB included knowledge; perceived susceptibility of OH diseases; perceived severity of OH diseases; cues to actions; expected social outcomes; perceived benefits of OHB; perceived barriers to OHB and self-efficacy. The adolescents’ and adults’ HBM-based questions were adopted mainly from the oral health behavior questionnaire for adolescents [37] and from other previous research [11, 38–41]. Questions about perceptions of OH care (Swiss healthcare system cares about my wellbeing; Swiss dental care system cares about my wellbeing) and autonomy (I feel that I am in control of the decisions related to my dental health) were added according to findings of the qualitative phase of the Axs2OH project. These constructs have been proposed as relevant context-specific extensions to the HBM in contexts where trust in institutions and perceived control influence health behaviors, particularly among marginalized populations [42–44]. All primary explanatory variables were self-reported during the in-person interview.

Potential confounders: Self-reported socio-demographic characteristics of participants considered as confounders based on *a priori* evidence included sex, age, and highest achieved educational level. Additional socio-demographic characteristics exhibiting only weak differences in their distribution between adequate and inadequate OHB were unlikely confounding reported associations between HBM-based explanatory variables and OHB. They were therefore merely used for describing the study population: residency permit type and year of arrival in Switzerland.

### Statistical Analysis

The participants’ characteristics are presented, stratified by OHB outcome variables, as mean and range for the numerical continuous variables, and as number and percentage for the categorical variables. Participants’ OHB is described as number and percentage with adequate toothbrushing, adequate interdental cleaning, or both. Differences in participant characteristics by toothbrushing and interdental cleaning behavior were examined descriptively using the Wilcoxon rank-sum test for continuous variables and Pearson’s chi-square test for categorical variables, with p-values reported for descriptive purposes only.

A multiphase approach consisting of 3 sequential phases was applied to examine the association between HBM-based variables with the two OHB outcome variables (toothbrushing and interdental cleaning). This phased approach was necessary due to the high number of original HBM-based explanatory variables investigated (k = 40) and the fact that they were in part correlated, in some cases despite belonging to different HBM domains. The multiphase approach additionally allowed identifying high polarity in the responses to some variables, which necessitated reconsidering their utility as targets to improve OHB. In summary, the goal of the sequential process over 3 phases was to identify HBM-relevant constructs and within these constructs specific variables and therefore questions which likely could have the biggest potential impact on ASRs OHB if asked by a dental professional.

#### Phase 1: Single Variable-Level Exploration of Association With OHB

This phase served as an initial exploration of the associations between outcome and exploratory variables and provided a useful reference for comparison with the final multivariable models in phase 3.

Responses to single HBM-based questions were mostly provided on a Likert scale. The distribution of answers to each HBM-based question is presented as domain-specific bar charts (Supplementary Figures S1–S12).

Regression analyses using generalized linear mixed-effects models with a binomial distribution and logit link, adjusting for age, sex, education, and household as a random effect, were conducted to initially investigate the association of binary OHBs with (a) each ordinal HBM-based explanatory variable separately, and (b) mutually adjusted ordinal HBM-based explanatory variables from the same domain (Supplementary Tables S1, S2).

#### Phase 2: Domain-Level Exploration of Association With OHB and Domain Selection

This phase aimed to identify the most influential HBM domains to subsequently prioritize domains and their specific questions in the explanatory variable selection in phase 3.

HBM-based explanatory variables could share underlying variance due to domain-specific overlaps (e.g., dental appearance included in benefits and severity domains). A Kendall’s tau ordinal association network was constructed to assess associations among ordinal HBM-based explanatory variables and identify potential conceptual overlaps across domains.

The first goal of this phase was to reduce multicollinearity, uncover latent constructs, and summarize each domain with fewer components. Given the ordinal metric of the HBM-based variables, it would have been inappropriate to use standard PCA, which assumes continuous variables with linear relationships among them. The risks of applying standard (linear) PCA to categorical and in particular ordinal variables has been highlighted in prior literature, for example, Linting et al. [45]. Among the possible alternatives, we decided to employ penalized non-linear PCA [46], which is flexible and able to capture the likely non-linearity of the relationship between ordinal responses in the same domain.

For each domain, the smallest number of principal components (PC) that together explained at least 50% of the total variance were included in the analysis. Knowledge domain was retained as ordinal variable due to the highly skewed response distribution that hindered the execution of PCA. The association of one or more of these PCs with OHB was tested in domain-specific models, using generalized linear mixed-effects models with a binomial distribution and logit link, adjusting for age, sex, education, and household. To explore potential non-linear associations, polynomial terms were included for each PC. There was minimal to no evidence of non-linear associations between the PCs and OHB outcome variables, and therefore polynomial terms were not retained further in the analysis (data not shown).

Domains whose PCs showed a p-value <0.05 for association with at least one of the two outcomes (toothbrushing or interdental cleaning) were retained for phase 3. Effect sizes from this phase’s models were not the focus of interpretation. Penalized non-linear PCA analysis has been carried out using *ordPCA* function from *ordPens* library in R.

#### Phase 3: Final Model on Most Influential HBM-Based Explanatory Variables of OHB

This phase served to identify the potentially most informative HBM-based explanatory variables for OHB. All the original ordinal HBM-based explanatory variables belonging to domains selected in phase 2 were retained in the current phase.

A step-wise selection (both backward and forward) was applied using the stepAIC function from the *MASS* package in R [47], guided by the Akaike Information Criterion (AIC) to retain the most potentially informative variables. The associations of the selected HBM-based explanatory variables (selected by the step-wise approach) with OHBs were tested in domain-specific models, employing generalized linear mixed-effects models with a binomial distribution and logit link, adjusting for age, sex, education, and household as random intercept.

Multicollinearity between the final selected HBM-based explanatory variables was assessed using generalized variance inflation factors (GVIF), which are appropriate for generalized linear mixed-effects models [48].

Data was complete for all variables and accordingly no imputation of information was needed. The data were analyzed by R (version 4.4.3). The level of significance was set to α = 0.05 and all estimates are reported with 95% confidence intervals (CI).

## Results

### Descriptive Statistics

Table 1 presents the demographic characteristics of the study sample (300 participants from 151 households), stratified by adequacy of toothbrushing and interdental cleaning. 95% of the study participants were Syrians (data not shown).

**TABLE 1:** Sociodemographic characteristics according to oral hygiene behavior. (Access to oral healthcare among asylum seekers and refugees, Switzerland, 2025).

Characteristic	Overall	Grouped by toothbrushing			Grouped by interdental cleaning		
	N = 300^a^ (100%)	Inadequate toothbrushing N = 142^b^ (47%)	Adequate toothbrushing N = 158^b^ (53%)	p-value^c^	Inadequate interdental cleaning N = 194^b^ (65%)	Adequate interdental cleaning N = 106^b^ *(35%)*	p-value^c^
Age	​	​	​	<0.001	​	​	0.2
Age in years	35 (14–89)	39 (14–89)	33 (14–70)	​	35 (14–89)	37 (14–70)	​
Sex	​	​	​	0.008	​	​	>0.9
Female	175 (58%)	71 (50%)	104 (66%)	​	113 (58%)	62 (58%)	​
Male	125 (42%)	71 (50%)	54 (34%)	​	81 (42%)	44 (42%)	​
Highest education level	​	​	​	0.028	​	​	0.012
Primary school (grades 1–6)	79 (26%)	47 (33%)	32 (20%)	​	62 (32%)	17 (16%)	​
Secondary school, middle school, or apprenticeship	162 (54%)	67 (47%)	95 (60%)	​	101 (52%)	61 (58%)	​
Technical college or university	45 (15%)	19 (13%)	26 (16%)	​	23 (12%)	22 (21%)	​
other	14 (4.7%)	9 (6.3%)	5 (3.2%)	​	8 (4.1%)	6 (5.7%)	​
Residency permit type	​	​	​	0.2	​	​	0.5
Provisional permit	78 (26%)	40 (28%)	38 (24%)	​	54 (28%)	24 (23%)	​
Refugee permit	168 (56%)	82 (58%)	86 (54%)	​	108 (56%)	60 (57%)	​
Work permit	54 (18%)	20 (14%)	34 (22%)	​	32 (16%)	22 (21%)	​
Year of arrival to Switzerland	​	​	​	0.8	​	​	0.005
Before 2015	73 (24%)	37 (26%)	36 (23%)	​	42 (22%)	31 (29%)	​
2015–2020	110 (37%)	49 (35%)	61 (39%)	​	62 (32%)	48 (45%)	​
2020–2022	75 (25%)	37 (26%)	38 (24%)	​	58 (30%)	17 (16%)	​
2023–2025	42 (14%)	19 (13%)	23 (15%)	​	32 (16%)	10 (9.4%)	​
Toothbrushing behavior	​	​	​	​	​	​	<0.001
Inadequate toothbrushing	142 (47%)	-	-	​	108 (56%)	34 (32%)	​
Adequate toothbrushing	158 (53%)	-	-	​	86 (44%)	72 (68%)	​
Interdental cleaning behavior	​	​	​	<0.001	​	​	​
Inadequate interdental cleaning	194 (65%)	108 (76%)	86 (54%)	​	-	-	​
Adequate interdental cleaning	106 (35%)	34 (24%)	72 (46%)	​	-	-	​

^a^ Adequate toothbrushing is toothbrushing at least twice a day versus less frequent. Adequate interdental cleaning is cleaning at least every second day versus less frequent.

^b^ Mean (Min–Max); n (%).

^c^ Wilcoxon rank sum test; Pearson’s Chi-squared test.

N: number.

53% of participants self-reported adequate toothbrushing behavior but the prevalence of adequate interdental cleaning was substantially lower (35%). Among participants with adequate toothbrushing, around 46% also practiced adequate interdental cleaning. Similarly, around 68% of participants with adequate interdental cleaning also brushed their teeth adequately.

Descriptively, adequate toothbrushing was more common among female and younger participants and participants with secondary or higher education levels, whereas adequate interdental cleaning was more common among participants with higher education and more years since arrival in Switzerland.

The distribution of single question answers by HBM domain is presented in Supplementary Figures S1–S12. In the knowledge domain, at least 93% of participants agreed or strongly agreed that smoking can affect OH and that sugary food and drinks negatively affect teeth. In the perception domain, only 19.7% strongly agreed that the dental system cares about their wellbeing, and over half disagreed or strongly disagreed with feeling in control of the decisions related to their dental health. In the susceptibility domain, at least 60% agreed or strongly agreed that they are susceptible to having dental caries or gum diseases. In the severity domain, 85% of participants considered being unable to eat their favorite foods due to oral diseases as serious or very serious, while 67% perceived being laughed at for OH issues as similarly serious. In the cues to action domain, the vast majority agree or strongly agree that parents (98%) and teachers (84%) have role model function in influencing OHB. In the expected social outcome domain, 69% of participants agreed or strongly agreed that people judge one another based on their teeth, while 96% emphasized the importance of fresh breath in socialization. In the benefits domain, 81.4% of participants agreed or strongly agreed that toothbrushing prevents oral diseases, compared to 59% for interdental cleaning. In the barriers domain, only 9% of participants agreed that they did not know how to brush their teeth, whereas 32% reported not knowing how to perform interdental cleaning. Additionally, 51% felt that their family did not encourage them to brush their teeth, and this figure rose to 68% for interdental cleaning. In the self-efficacy domain, self-efficacy under stress or time pressure was high for toothbrushing (more than 38% quite to very confident), but very low for interdental cleaning (70% not confident).

#### Phase 1: Single Variable-Level Exploration of Association With OHB

Explanatory variables related to the perception of autonomy, to selected benefits and selected barriers and to self-efficacy showed potential association with OHB in this phase (Supplementary Tables S1, S2). Specifically, feeling in control on decisions related to ones OH was associated with a higher likelihood of toothbrushing (OR_mutually adjusted within domain_ = 1.23, 95% CI 1.00–1.51) and suggestively also with a higher likelihood of interdental cleaning (OR_mutually adjusted within domain_ = 1.15, 95% CI 0.94–1.40). Specifically, a high-level confidence in toothbrushing when under time pressure was associated with a higher likelihood of conducting toothbrushing (OR_mutually adjusted within domain_ = 2.38, 95% CI 1.40–4.05) and suggestively also with a higher likelihood of interdental cleaning (OR_mutually adjusted within domain_ = 1.49, 95% CI 0.86–2.56). Not knowing how to perform the respective OHB was a strong barrier to interdental cleaning (OR_mutually adjusted within domain_ = 0.68, 95% CI 0.56–0.82), but not to toothbrushing (OR_mutually adjusted within domain_ = 0.97, 95% CI 0.69–1.37).

#### Phase 2: Domain-Level Exploration of Association With OHB and Domain Selection

Kendall’s tau ordinal associations between the potential HBM-based explanatory variables are visualized in Supplementary Figure S13. Strong ordinal associations clustered within domains, particularly among self-efficacy, barriers, and benefits variables, thus giving justification to the PCA approach for selecting HBM domains in phase 2. HBM-based explanatory variables that are specific for each OHB, tended to be associated more strongly within rather than across the two behaviors.

Results on the age-, sex-, education-, and household adjusted associations of domain-specific PCs with adequate toothbrushing and interdental cleaning, respectively are presented in Tables 2, 3. Knowledge was retained in the model as ordinal variables given its strong skewedness.

**TABLE 2:** Domain-specific association^a^ of principal components with adequate toothbrushing^b^, adjusted for age, sex, education and household as random effect. (Access to oral healthcare among asylum seekers and refugees, Switzerland, 2025).

Domain	Principal component^c^/variable	OR	95% CI	P-value
Perceptions	per_pc1	1.39	1.09, 1.78	0.008
Knowledge^d^	Knowledge sugar	0.99	0.63, 1.57	>0.900
	Knowledge smoke	1.04	0.70, 1.54	0.800
Susceptibility	sus_pc1	0.80	0.64, 1.00	0.047
Severity	sev_pc1	1.06	0.88, 1.28	0.500
	sev_pc2	0.88	0.67, 1.15	0.400
	sev_pc3	0.82	0.61, 1.12	0.200
Cues to action	cue_pc1	1.05	0.80, 1.37	0.700
	cue_pc2	0.92	0.66, 1.27	0.600
Expected social outcomes	expct_pc1	1.07	0.83, 1.39	0.600
	expct_pc2	0.8	0.58, 1.10	0.200
Benefit of toothbrushing	ben_brush_pc1	1.08	0.90, 1.29	0.400
	ben_brush_pc2	0.88	0.65, 1.20	0.400
Barriers of toothbrushing	bar_brush_pc1	0.28	0.15, 0.51	<0.001
	bar_brush_pc2	1.02	0.70, 1.47	>0.900
	bar_brush_pc3	1.81	1.19, 2.76	0.006
Self-efficacy	eff_brush_pc1	7.87	2.15, 28.90	0.002

^a^ Each domain-specific model includes all principal components from the respective domain.

^b^ Adequate toothbrushing is toothbrushing at least twice a day versus less frequent.

^c^ Meaning of principal components based on the loadings (see Supplementary Table S3).

per_pc1: High positive perceptions.

sus_pc1: High susceptibility of oral health disease.

sev_pc1: High perceived seriousness of oral health diseases and connection to overall health.

sev_pc2: High perceived severity of tooth loss and oral health diseases impacting on heath, with low seriousness of oral health disease and functions.

sev_pc3: High severity due to being laughed at based on oral health condition.

ben_brush_pc1: High benefits of toothbrushing.

ben_brush_pc2: High perceived preventive benefit of toothbrushing with less emphasis on sensory benefits.

bar_brush_pc1: High barriers to toothbrushing.

bar_brush_pc2: High social support/knowledge barriers, but not painful.

bar_brush_pc3: High knowledge on toothbrushing and social support with low motivation.

cue_pc1: High cues.

cue_pc2: High remind family/friend and low cues from teachers.

expct_pc1: High social value of oral health.

expct_pc2: High fear of judgment with low value of cleanness/aesthetics.

eff_brush_pc1: High self-efficacy with toothbrushing.

^d^ Knowledge was retained as original ordinal explanatory variable due to the skewedness of responses, which hindered the principal component analysis.

OR: Odds ratios. CI: Confidence intervals. PC: principal component.

**TABLE 3:** Domain-specific association^a^ of principal components with adequate interdental cleaning^b^, adjusted for age, sex, education and household as random effect. (Access to oral healthcare among asylum seekers and refugees, Switzerland, 2025).

Domain	Principal component^c^/variable	OR	95% CI	P-value
Perceptions	per_pc1	1.19	0.94, 1.50	0.200
Knowledge^d^	Knowledge sugar	1.81	1.03, 3.20	0.040
	Knowledge smoke	1.08	0.70, 1.66	0.700
Susceptibility	sus_pc1	0.92	0.74, 1.14	0.400
Severity	sev_pc1	1.03	0.85, 1.24	0.800
	sev_pc2	0.85	0.66, 1.11	0.200
	sev_pc3	0.77	0.57, 1.04	0.085
Cues to action	cue_pc1	0.95	0.73, 1.24	0.700
	cue_pc2	0.89	0.65, 1.23	0.500
Expected social outcomes	expct_pc1	0.85	0.67, 1.08	0.200
	expct_pc2	0.85	0.63, 1.15	0.300
Benefits of interdental cleaning	ben_interdent_pc1	1.44	1.17, 1.78	<0.001
Barriers of interdental cleaning	bar_interdent_pc1	0.75	0.63, 0.89	0.001
	bar_interdent_pc2	0.97	0.77, 1.22	0.800
	bar_interdent_pc3	0.79	0.61, 1.03	0.088
Self-efficacy	eff_interdent_pc1	2.96	2.14, 4.09	<0.001

^a^ Each domain-specific model includes all principal components from the respective domain.

^b^ Adequate interdental cleaning is cleaning at least every second day versus less frequent.

^c^ Meaning of principal components based on the loadings (see Supplementary Table S3).

per_pc1: High positive perceptions.

sus_pc1: High susceptibility of oral health disease.

sev_pc1: High perceived seriousness of oral health diseases and connection to overall health.

sev_pc2: High perceived severity of tooth loss and oral health diseases impact on heath, with low seriousness of oral health disease and functions.

sev_pc3: High severity due to being laughed at based on oral health condition.

ben_interdent_pc1: High benefits of interdental cleaning.

bar_interdent_pc1: High barriers to interdental cleaning.

bar_interdent_pc2: High forgetting as a barrier but low pain and bleed.

bar_interdent_pc3: High knowledge and social support barriers with low pain and bleed.

cue_pc1: High cues.

cue_pc2: High remind family/friend and low cues from teachers.

expct_pc1: High social value of oral health.

expct_pc2: High fear of judgment with low value of cleanness/aesthetics.

eff_interdent_pc1: High self-efficacy with interdental cleaning.

^d^ Knowledge was retained as original ordinal explanatory variable due to the skewedness of responses, which hindered the principal component analysis.

OR: Odds ratios. CI: Confidence intervals. PC: principal component.

As the goal of this phase was domain selection rather than inference, effect estimates and p-values are reported for transparency but were not interpreted. Domains that passed the selection phase (association with at least one OHB outcome variable at p-value <0.05) to be considered in phase 3 included perceptions, knowledge, susceptibility, benefits, barriers, and self-efficacy. However, susceptibility was no longer considered due to the issue of potential bidirectional association with the outcomes (e.g., higher perceived susceptibility to get caries might lead to more adequate OHB or performing adequate OHB might lead to lower perceived susceptibility of getting caries). Knowledge was no longer considered as the highly polarized response distribution limits the utility of these questions for improving OHB (Supplementary Figure S2).

Accordingly, domains perceptions, benefits, barriers and self-efficacy were taken forward to phase 3.

#### Phase 3: Final Model on Most Influential HBM-Based Explanatory Variables of OHB

All original ordinal HBM-based variables belonging to domains retained in phase 2 (perceptions, benefits, barriers, and self-efficacy) were entered into a step-wise selection process to identify the most informative single explanatory variables of the OHBs based on the AIC as described in statistical methods. Then the selected HBM-based variables were used as explanatory variables in the final models for the two OHB outcomes.

#### Toothbrushing as Outcome

Table 4 summarizes the final multivariable generalized linear mixed-effects model (binomial distribution, logit link) examining determinants of adequate toothbrushing as outcome.

**TABLE 4:** Influential^a^ Health Belief Model determinants of adequate toothbrushing^b^. (Access to oral healthcare among asylum seekers and refugees, Switzerland, 2025).

Domain	Specific questions		OR^c^	95% CI	P-value
Perceptions	How much do you agree with the following^d^	I feel that I am in control of the decisions related to my dental health	1.40	0.91, 2.17	0.13
Benefit of toothbrushing	How much do you agree with the following^d^	My mouth feels better after I brush my teeth	0.59	0.28, 1.23	0.20
Barriers of toothbrushing	How much do you agree with the following^d^	Toothbrushing is painful	0.57	0.24, 1.39	0.20
		I don’t have time to brush my teeth at least two times a day	0.57	0.24, 1.35	0.20
		I forget to brush at least two times a day	0.69	0.36, 1.31	0.30
Self-efficacy	How confident are you that you will brush your teeth for 2 min twice daily on the circumstances below^e^	When you don’t have time	1.97	0.51, 7.66	0.30
		When you are under a lot of stress	2.93	0.68, 12.7	0.20
		When you are anxious	1.77	0.87, 3.59	0.12

^a^ Influential domains and variables therein were identified in the context of:

1- domain-specific models with principal components as domain-specific determinants (see Table 2).

2- considering domains and their original variables if the domain reached statistical significance at p < 0.05 in [1] for at least one of two oral hygiene behavior outcomes.

3- based on an adjusted model containing all the domains selected in [2] and the original variables therein and applying a stepwise selection (both backward and forward) using the *stepAIC* function from the *MASS* package in R [47] to identify the most informative single variables as predictors of OHB, based on AIC.

^b^ Adequate toothbrushing is toothbrushing at least twice a day versus less frequent.

^c^ Adjusted for sex, age in years, education and household ID as random effect.

^d^ The answer is an agreement 5-point Likert scale (see Questionnaire in Supplementary Material 1).

^e^ The answer is a confidence 5-point Likert scale (see Questionnaire in Supplementary Material 1).

OR: Odds ratios. CI: Confidence intervals. AIC: akaike information criterion.

The final model retained the following domains and key HBM-based explanatory variables of adequate or inadequate toothbrushing: a) self-efficacy: high levels of confidence that one will brush for 2 min twice a day under lack of time (OR = 1.97, 95% CI 0.51–7.66); being stressed (OR = 2.93, 95% CI 0.68–12.70); or being anxious (OR = 1.77, 95% CI 0.87–3.59) is associated with higher likelihood of adequate toothbrushing; b) barriers to toothbrushing: reporting toothbrushing as painful (OR = 0.57, 95% CI 0.24–1.39); not having time (OR = 0.57, 95% CI 0.24–1.35) or forgetting to adequately brush (OR = 0.69, 95% CI 0.36–1.31) is associated with lower likelihood of adequate toothbrushing, c) benefits of toothbrushing: high agreement with one’s mouth feeling better after toothbrushing teeth (OR = 0.59, 95% CI 0.28–1.23) is associated with lower likelihood of adequate toothbrushing, and d) autonomy: high perceived control of one’s control of decisions related to dental health is associated with higher likelihood of adequate toothbrushing (OR = 1.40, 95% CI 0.91–2.17).

The findings from the final model in Table 4 were largely consistent in direction with the domain-specific single variable models presented in Supplementary Table S1, except for one’s mouth feeling better after toothbrushing which showed no association, in agreement with the lacking association of PCs related to benefits showing no association with toothbrushing in phase 2.

#### Interdental Cleaning as Outcome

Table 5 results from analyses equivalent to Table 4, but for interdental cleaning as outcome.

**TABLE 5:** Influential^a^ Health Belief Model determinants of adequate interdental cleaning^b^. (Access to oral healthcare among asylum seekers and refugees, Switzerland, 2025).

Domain	Specific questions		OR^c^	95% CI	P-value
Benefits of interdental cleaning	How much do you agree with the following^d^	My mouth feels better after I clean the spaces between my teeth	1.61	1.14, 2.27	0.007
		My breath is fresher after I clean the spaces between my teeth	0.69	0.49, 0.96	0.029
		Cleaning the spaces between my teeth at least once a day will save me money on dental expenses	1.36	1.01, 1.82	0.041
Barriers of interdental cleaning	How much do you agree with the following^d^	I do not know how to clean the spaces between my teeth properly	0.76	0.61, 0.95	0.014
		Dental floss/tools for cleaning between my teeth is expensive	1.25	0.95, 1.67	0.120
		My teeth will break when I clean between my teeth	0.80	0.59, 1.07	0.130
		I feel that my family didn’t encourage me to clean between my teeth regularly	1.17	0.94, 1.45	0.200
Self-efficacy	How confident are you that you will clean between your teeth once a day on the circumstances below^e^	When you are under a lot of stress	3.10	2.28, 4.22	<0.001

^a^ Influential domains and variables therein were identified in the context of:

1- domain-specific models with principal components as domain-specific determinants (see Table 2).

2- considering domains and their original variables if the domain reached statistical significance at p < 0.05 in [1] for at least one of two oral hygiene behavior outcomes.

3- based on an adjusted model containing all the domains selected in [2] and the original variables therein and applying a stepwise selection (both backward and forward) using the *stepAIC* function from the *MASS* package in R [47] to identify the most informative single variables as predictors of OHB, based on AIC.

^b^ Adequate interdental cleaning is cleaning at least every second day versus less frequent.

^c^ Adjusted for sex, age in years, education and household ID as random effect.

^d^ The answer is an agreement 5-point Likert scale (see Questionnaire in Supplementary Material 1).

^e^ The answer is a confidence 5-point Likert scale (see Questionnaire in Supplementary Material 1).

OR: Odds ratios. CI: Confidence intervals. AIC: akaike information criterion.

The following domains and variables were retained in the final model as most influential to identify adequate and inadequate interdental cleaning: a) self-efficacy: high confidence in conducting interdental cleaning when under stress (OR = 3.10, 95% CI 2.28–4.22) is associated with higher likelihood of adequate interdental cleaning, b) benefits of interdental cleaning: agreeing to interdental cleaning making mouth feel better (OR = 1.61, 95% CI 1.14–2.27) and with saving money on dental expenses (OR = 1.36, 95% CI 1.01–1.82) is associated with higher likelihood of adequate interdental cleaning, whereas agreeing with breath being fresher (OR = 0.69, 95% CI 0.49–0.96) is associated with lower likelihood of adequate interdental cleaning; c) barriers to interdental cleaning: they are associated with the likelihood of adequate interdental cleaning in different directions, e.g., agreeing to interdental cleaning don’t know how (OR = 0.76, 95% CI 0.61–0.95), being expensive (OR = 1.25, 95% CI 0.95–1.67), causing teeth breaking (OR = 0.80, 95% CI 0.59–1.07), not encouraged by family (OR = 1.17, 95% CI 0.94–1.45). The direction of associations in the final model shown in Table 5 was largely consistent with the domain-specific single variable models (Supplementary Table S2).

Multicollinearity diagnostics indicated little to no concern regarding collinearity among the final selected explanatory variables for both final models, with all adjusted GVIF values below commonly used thresholds for problematic multicollinearity (Supplementary Tables S5, S6).

## Discussion

Results of this cross-sectional study among ASRs might point to substantial levels of OHB not meeting OH guidelines [49]. Only half the participants reported adequate toothbrushing and even less (about a third) adequate interdental cleaning. Agreement on the benefits and self-confidence of toothbrushing was generally higher for toothbrushing than interdental cleaning, indicating that the latter remains a less familiar OHB.

Given that toothbrushing and flossing in the general Swiss population are influenced by income and education [50], ASRs likely face additional structural and personal barriers that limit preventive OH care (check-ups, hygiene instruction, costs for interdental cleaning instruments). Access to services often depends on permit type and cantonal guidelines, which may restrict preventive services for those receiving social welfare [51–54].

To plan effective public dental health interventions, it is important to understand the OH-related attitudes/beliefs [9, 11], especially among ASRs. The current study identified relevant and influential HBM-based constructs and within these constructs explanatory variables influencing OHB among ASRs. The results point to priority target domains and variables and questions to be asked in clinical settings for testing in intervention studies and could ultimately support the planning of effective preventive services for ASRs.

### Self-Efficacy

Self-efficacy (the confidence in one’s ability to successfully perform a specific behavior) is a central determinant of health behavior and a crucial factor in motivating individuals to take an active role in their health. It strongly influences the maintenance of self-management and the adoption of healthy practices [55], including OHB [56].

Self-efficacy in the current study was characterized by having the confidence in adequate toothbrushing and interdental cleaning even in the face of having time constraints or being stressed and anxious was by far most strongly and consistently associated with both OHBs.

These findings on self-efficacy with both OHBs are somewhat parallel to Jönsson et al. work on the effect of individually tailored OHB promotion programs, following a cognitive behavioral approaches (incorporating practical and theoretical knowledge, motivation, self-monitoring and maintenance of the behavior), on the amount of dental plaque that is accumulated on and between the teeth [57].

### Autonomy and Brushing Only

The final analyses suggested that perceived autonomy on own OH could play a significant role in shaping toothbrushing, but not interdental cleaning practices. Specifically, ASRs who felt more in control of their OH decisions had higher odds of toothbrushing adequately (at least twice a day). This aligns with previous evidence highlighting the role of autonomy in health-promoting behaviors [58].

### Benefits

Established health habits are determined by a profound belief in their benefits [10].

The perceived benefits domain only mattered for interdental cleaning, but not toothbrushing. In fact unexpectedly, this study’s results suggested that perceiving toothbrushing as making the mouth feel better, was associated with lower odds of adequate toothbrushing, reflecting a disconnection between belief/attitude and behavior (e.g., individuals agreeing with this statement but not translating it into action due to some barriers).

In contrast, regarding adequate interdental cleaning (at least each other day), the domain of perceived benefits in general, and several perceived specific benefits were positively associated with adequate practice. Believing that interdental cleaning makes the mouth feel better (unlike toothbrushing) or reduces future dental treatment costs was associated with higher odds of adequate cleaning. Surprisingly on benefits, participants who agreed that interdental cleaning results in fresher breath were less likely to adopt it.

The finding that some perceived benefits of the two OHBs were negatively associated with adequately adopting the respective OHB could be explained by Brown et al.’s conclusion on health behaviors needing self-regulatory skills to be translated into actions [59]. However, this gap between belief/attitude and behavior may be further exacerbated by persistent barriers, which interfere with the ability to act on positive beliefs.

In summary, the evidence on benefits as particular target for promoting OHB was inconsistent and rather weak.

### Barriers

In contrast to perceived benefits, the perceived barrier domain was inversely associated with both OHBs, toothbrushing and interdental cleaning.

Barriers as perceiving toothbrushing being painful, lack of time, and forgetfulness were found associated with reduced odds of toothbrushing adequately. Among the reported barriers to interdental cleaning, not knowing how to clean between the teeth and fearing tooth breakage were both negatively associated with adequate interdental cleaning.

These barriers to OHBs among ASRs could occur due to OH care and OHB experienced in the home country [60, 61] or the accumulation of migration-related stressors. ASRs are subjected to trauma-related mental health issues, rooted in the hardships encountered before the decision of migration and along the migration route, including collective survival concerns, precarious living conditions, and prolonged uncertainty [19]. After migration, mental health challenges are often exacerbated by uncertainty surrounding legal status and social disruption, including loss of community bonds, financial worries, unfamiliar cultural norms and limited access to social support systems [20].

As a result, ASRs may experience reduced financial resources as well as motivation or energy to maintain regular OHBs, particularly in the absence of pre-established health habits, limited OH knowledge, or negative experiences with OH care in their country of origin [21, 22].

Interestingly on barriers to interdental cleaning, participants who reported that interdental cleaning tools are expensive or lacked family encouragement were more likely to clean adequately. It is likely that participants who conduct interdental cleaning, have already purchased the necessary tools, are therefore more aware of their cost than those who do not. At the same time, reporting insufficient family support might reflect a stronger personal conviction about the importance of the habit, which could in turn promote more consistent practice [62].

### Strengths and Limitations

One of the strengths of this study lies in its multidimensional and theory-driven approach, additionally informed by an embedded qualitative study, as well as its focus on a highly under-researched population. However, its cross-sectional design hinders causal inference. This may have been particularly true for the susceptibility domain, which we therefore excluded in the final variable selection step. The high observed level of knowledge related to sugar and smoking being bad for teeth limited our ability to investigate its potential as target to improve OHB in populations less knowledgeable. Self-reported data harbors the risk of recall and social desirability biases.

Additionally, the sample may not be fully representative of all Arabic-speaking ASRs in Switzerland given that different regions have different health and OH care policies for ASRs. The use of snowball sampling, while necessary for recruitment, further limits generalizability and may introduce bias in both prevalence estimates and confidence intervals.

Finally, due to limited resources and the exploratory nature of the study, we were unable to formally validate the questionnaire in this specific population. Yet, many questions were derived from instruments validated in other populations. In addition, the comprehensibility of the questions was piloted beforehand in the target population. Finally, the large number of questions asked across different HBM-domains combined with the three-phased approach allowed prioritizing constructs and within the constructs variables for further testing.

The current observational study and its limitations point to the fact that the study is hypothesis generating. The priority domains and variables suggested as being relevant in determining OHB in ASRs need further testing in intervention studies that allow assessing causality.

### Conclusion

The results suggest that the relevance of promoting OHB in ASRs, a health aspect with potentially broad and beneficial consequences, is high. Self-efficacy, autonomy in decision-making, and selected barriers and benefits might be critical intervention targets with a potential to positively influence OHB. If further confirmed in randomized trials, the results could encourage dental professionals to assess patients’ confidence (self-efficacy), perceived autonomy, and beliefs around benefits and barriers to oral hygiene. Policymakers could strengthen migrant health packages by including OH education and funding preventive services. School-based programs targeting migrant children and their families, as well as integration services offering dental hygiene education and screening, may support long-term improvements.
