# Supplementary material for: Oral Hygiene Behavior Among Asylum Seekers and Refugees Using Health Beliefs Model: A Cross-Sectional Study
Source: Int J Public Health. 2026 Feb 23;71:1609334. doi: 10.3389/ijph.2026.1609334 (PMC12968041; doi:10.3389/ijph.2026.1609334)
Supplement: Supplementary file 1 [file DataSheet1.pdf]

Journal name: International journal of public health

Article title: Oral hygiene behavior among asylum seekers and refugees using Health Beliefs Model: A Cross-Sectional Study

Supplementary material

Supplementary Table S1

Table S1. Association of ordinal variables with adequate toothbrushing[a], adjusted for age, sex, education, and household as random effect

Domain-specific questions	Adjusted association at individual variable level<br>Model 1[b]	Additionally mutually adjusted association within domain<br>Model 2[c]
	OR (95% CI)	OR (95% CI)
Perception[d]		
Swiss healthcare system cares about my well-being	1.18 (0.89, 1.56)	0.99 (0.72, 1.36)
Swiss dental care system cares about my well-being	1.35 (1.05, 1.73)	1.27 (0.96, 1.67)
I feel that I am in control of the decisions related to my dental health	1.28 (1.05, 1.56)	1.23 (1.00, 1.51)
Knowledge[d]		
Smoking can affect oral health	1.04 (0.71, 1.53)	1.04 (0.70, 1.54)
Sugary food and drinks affect teeth	1.01 (0.65, 1.58)	0.99 (0.63, 1.57)
Susceptibility[d]		
There is a chance that I will get caries.	0.84 (0.68, 1.05)	0.93 (0.72, 1.20)
There is a chance that I will have periodontal disease.	0.79 (0.64, 0.98)	0.81 (0.63, 1.03)
My mouth is in bad condition.	0.95 (0.76, 1.18)	1.04 (0.82, 1.32)
Severity		
I will lose my teeth if I get tooth decay or gum disease[d]	1.03 (0.83, 1.28)	0.96 (0.76, 1.22)
Tooth decay and gum diseases can cause other health problems[d]	1.17 (0.92, 1.49)	1.15 (0.89, 1.49)
My self-consciousness will be impaired if I have poor oral health conditions[d]	1.05 (0.71, 1.54)	0.93 (0.60, 1.43)
If I have caries, for me that is …[e]	1.20 (0.89, 1.61)	1.15 (0.79, 1.67)
If I have gum or periodontal disease, for me that is … [e]	1.27 (0.94, 1.72)	1.19 (0.83, 1.73)
If my teeth do not look good because of oral diseases, for me that is … [e]	1.13 (0.78, 1.63)	1.06 (0.69, 1.61)
If I can't eat my favorite food because of oral diseases, for me that is … [e]	0.84 (0.63, 1.13)	0.74 (0.53, 1.04)
If I get laughed at by friends or relatives/classmates because of oral diseases, for me that is … [e]	1.05 (0.87, 1.27)	1.06 (0.86, 1.29)
Cues to action[d]		
My parents/ friends often remind me of brushing and interdental cleaning	0.98 (0.82, 1.16)	0.97 (0.81, 1.16)
Teachers often remind their students of brushing and interdental cleaning	1.03 (0.77, 1.38)	1.03 (0.77, 1.38)
Parents should help their kids (up to 6 years old) to brush properly	1.28 (0.52, 3.14)	1.27 (0.51, 3.14)
Expected social outcomes[d]		
People judge each other on the basis of their teeth	0.93 (0.75, 1.15)	0.90 (0.72, 1.13)
I appreciate it when people with whom I socialize have well-maintained teeth	1.24 (0.93, 1.64)	1.25 (0.94, 1.67)

In social contacts, oral health with fresh breath is important	1.10 (0.65, 1.85)	1.02 (0.59, 1.75)
Benefits of brushing[d]		
Brushing my teeth at least two times a day will prevent tooth decay and gum disease	1.03 (0.80, 1.34)	1.01 (0.73, 1.39)
If I brush my teeth at least two times a day they will last a lifetime	0.97 (0.78, 1.20)	0.88 (0.67, 1.15)
My mouth feels better after I brush them	1.10 (0.77, 1.58)	0.97 (0.62, 1.51)
My breath feels better after I brush them	1.22 (0.82, 1.82)	1.21 (0.75, 1.95)
Brushing my teeth at least two times a day will save me money on dental expenses	1.09 (0.86, 1.38)	1.09 (0.81, 1.45)
My teeth will look better if I brush at least two times a day	1.17 (0.88, 1.54)	1.14 (0.82, 1.58)
Barriers to brushing[d]		
Tooth brushing is painful	0.66 (0.52, 0.83)	0.74 (0.53, 1.03)
My teeth will break when I brush	0.94 (0.81, 1.08)	0.88 (0.62, 1.24)
My gums will bleed when I brush	0.63 (0.51, 0.78)	0.76 (0.58, 1.00)
I forget to brush at least two times a day	0.58 (0.47, 0.72)	0.67 (0.50, 0.88)
If I am tired I don’t brush my teeth	0.50 (0.38, 0.67)	0.63 (0.45, 0.87)
Toothpaste is expensive	0.91 (0.71, 1.16)	0.87 (0.62, 1.22)
I don’t have time to brush my teeth at least two times a day	0.61 (0.46, 0.81)	0.79 (0.56, 1.10)
I feel that my family didn’t encourage me to brush my teeth regularly	0.94 (0.79, 1.12)	1.01 (0.80, 1.27)
I don’t know how to brush my teeth properly	0.84 (0.64, 1.09)	0.97 (0.69, 1.37)
Self-efficacy with brushing[f]		
When you don’t have time	4.38 (2.56, 7.51)	2.38 (1.40, 4.05)
When you are under a lot of stress	4.30 (2.34, 7.89)	1.66 (0.98, 2.80)
When you are anxious	3.43 (2.38, 4.93)	1.85 (1.16, 2.95)

[a] Adequate brushing is brushing at least twice a day versus less frequent. Number of participants with adequate toothbrushing / those with inadequate toothbrushing: 158 / 142

[b] Model 1: separate models for individual variables

[c] Model 2: separate models for domains, mutually adjusting for variables in the specific domain

[d] To what extent do you agree: (strongly disagree; disagree; neutral; agree; strongly agree)

[e] Please select what applies to you: (not serious; a little serious; partially serious; serious; very serious)

[f] How confident are you that you will brush your teeth for 2 min twice daily on the circumstances below? (not confident; a bit confident; fairly confident; quite confident; very confident)

OR: Odds Rations. CI: Confidence Intervals.

Supplementary Table S2

Table S2. Association of ordinal variables with adequate interdental cleaning[a], adjusted for age, sex, education, and household as random effect

Domain-specific questions	Adjusted association at individual variable level Model 1[b]	Additionally mutually adjusted association within domain Model 2[c]
	OR (95% CI)	OR (95% CI)
Perception[d]		
Swiss healthcare system cares about my well-being	1.11 (0.83, 1.48)	1.01 (0.74, 1.39)
Swiss dental care system cares about my well-being	1.16 (1.16, 1.17)	1.11 (0.85, 1.44)
I feel that I am in control of the decisions related to my dental health	1.17 (0.96, 1.43)	1.15 (0.94, 1.40)
Knowledge[d]		
Smoking can affect oral health	1.21 (0.80, 1.82)	1.08 (0.70, 1.66)
Sugary food and drinks affect teeth	1.83 (1.05, 3.20)	1.81 (1.03, 3.20)
Susceptibility[d]		
There is a chance that I will get caries.	0.99 (0.80, 1.22)	0.98 (0.77, 1.25)
There is a chance that I will have periodontal disease.	1.01 (0.82, 1.24)	1.03 (0.81, 1.31)
My mouth is in bad condition.	0.97 (0.78, 1.21)	0.97 (0.76, 1.22)
Severity		
I will lose my teeth if I get tooth decay or gum disease[d]	1.08 (0.86, 1.34)	1.06 (0.83, 1.34)
Tooth decay and gum diseases can cause other health problems[d]	1.02 (0.82, 1.28)	1.00 (0.78, 1.30)
My self-consciousness will be impaired if I have poor oral health conditions[d]	0.98 (0.67, 1.42)	0.94 (0.62, 1.44)
If I have caries, for me that is …[e]	1.19 (0.87, 1.62)	1.07 (0.73, 1.58)
If I have gum or periodontal disease, for me that is …[e]	1.32 (0.95, 1.84)	1.33 (0.90, 1.97)
If my teeth do not look good because of oral diseases, for me that is …[e]	0.89 (0.63, 1.26)	0.80 (0.53, 1.22)
If I can't eat my favorite food because of oral diseases, for me that is …[e]	1.02 (0.77, 1.36)	1.05 (0.76, 1.45)
If I get laughed at by friends or relatives/classmates because of oral diseases, for me that is…[e]	0.91 (0.76, 1.09)	0.90 (0.74, 1.10)
Cues to action[d]		
My parents/ friends often remind me of brushing and interdental cleaning	0.98 (0.82, 1.16)	0.99 (0.83, 1.18)
Teachers often remind their students of brushing and interdental cleaning	0.77 (0.58, 1.02)	0.75 (0.56, 0.99)
Parents should help their kids (up to 6 years old) to brush properly	2.01 (0.70, 5.81)	2.36 (0.79, 7.03)
Expected social outcomes[d]		
People judge each other on the basis of their teeth	0.89 (0.72, 1.11)	0.90 (0.72, 1.12)
I appreciate it when people with whom I socialize have well-maintained teeth	1.08 (0.82, 1.42)	1.15 (0.87, 1.53)

In social contacts, oral health with fresh breath is important	0.71 (0.43, 1.16)	0.67 (0.40, 1.13)
Benefit of interdental cleaning[d]		
Cleaning the spaces between my teeth at least once a day will prevent tooth decay and gum disease	1.59 (1.24, 2.03)	1.20 (0.87, 1.65)
If I clean the spaces between my teeth at least once a day they will last a lifetime	1.48 (1.18, 1.85)	1.06 (0.80, 1.41)
My mouth feels better after I clean the spaces between my teeth	1.84 (1.45, 2.32)	1.74 (1.31, 2.33)
My breath is fresher after I clean the spaces between my teeth	1.42 (1.14, 1.78)	0.86 (0.64, 1.15)
Cleaning the spaces between my teeth at least once a day will save me money on dental expenses	1.57 (1.25, 1.97)	1.20 (0.88, 1.63)
My mouth will look better if I clean the spaces between my teeth once a day	1.35 (1.11, 1.65)	1.00 (0.79, 1.27)
Barriers to interdental cleaning[d]		
Interdental cleaning is painful	0.81 (0.66, 1.00)	0.89 (0.69, 1.14)
My teeth will break when I clean between my teeth	0.73 (0.58, 0.93)	0.83 (0.64, 1.06)
My gums will bleed when I clean between my teeth	0.85 (0.70, 1.02)	1.05 (0.84, 1.33)
I forget to clean between my teeth at least once a day	0.90 (0.75, 1.09)	1.08 (0.87, 1.34)
If I am tired I don’t clean between my teeth	0.72 (0.60, 0.86)	0.76 (0.62, 0.92)
Dental floss/tools for cleaning between my teeth is expensive	0.92 (0.72, 1.17)	1.07 (0.85, 1.36)
I don’t have time to clean between my teeth at least once a day	0.70 (0.55, 0.91)	0.78 (0.59, 1.02)
I feel that my family didn’t encourage me to clean between my teeth regularly	0.94 (0.78, 1.13)	1.08 (0.90, 1.30)
I do not know how to clean the spaces between my teeth properly	0.63 (0.52, 0.77)	0.68 (0.56, 0.82)
Self-efficacy with interdental cleaning[f]		
When you don’t have time	3.27 (2.29, 4.65)	1.49 (0.86, 2.56)
When you are under a lot of stress	3.48 (2.41, 5.02)	1.46 (0.61, 3.53)
When you are anxious	3.59 (2.45, 5.27)	1.78 (0.80, 3.95)

[a] Adequate interdental cleaning is cleaning at least every second day versus less frequent. Number of participants with adequate interdental cleaning / those with inadequate interdental cleaning: 106 / 194
[b] Model 1: separate models for individual variables
[c] Model 2: separate models for domains, mutually adjusting for variables in the specific domain
[d] To what extent do you agree (strongly disagree; disagree; neutral; agree; strongly agree)
[e] Please select what applies to you: (not serious; a little serious; partially serious; serious; very serious)
[f] How confident are you that you will clean between your teeth once a day on the circumstances below? (not confident; a bit confident; fairly confident; quite confident; very confident)
OR: Odds Rations. CI: Confidence Intervals.

Supplementary Table S3

Table S3. Principal component loadings[a] for Health Belief Model-based determinants variables

Domain and variable	Principal component 1	Principal component 2	Principal component 3
Perception[b]			
Swiss healthcare system cares about my well-being	0.64	-	-
Swiss dental care system cares about my well-being	0.55	-	-
I feel that I am in control of the decisions related to my dental health	0.53	-	-
Knowledge[b]			
Smoking can affect oral health	-	-	-
Sugary food and drinks affect teeth	-	-	-
Susceptibility[b]			
There is a chance that I will get caries.	0.59	0.48	-0.65
There is a chance that I will have periodontal disease.	0.62	0.24	0.75
My mouth is in bad condition.	0.51	-0.84	-0.15
Severity			
I will lose my teeth if I get tooth decay or gum disease[b]	0.23	-0.53	-0.01
Tooth decay and gum diseases can cause other health problems[b]	0.37	-0.49	-0.03
My self-consciousness will be impaired if I have poor oral health conditions[b]	0.35	-0.41	0.02
If I have caries, for me that is …[c]	0.40	0.28	0.49
If I have gum or periodontal disease, for me that is …[c]	0.41	0.29	0.45
If my teeth do not look good because of oral diseases, for me that is …[c]	0.42	0.12	-0.22
If I can’t eat my favorite food because of oral diseases, for me that is …[c]	0.35	0.28	-0.35
If I get laughed at by friends or relatives/classmates because of oral diseases, for me that is…[c]	0.25	0.25	-0.63
Cues to action[b]			
My parents/ friends often remind me of brushing and interdental cleaning	0.49	0.84	-
Teachers often remind their students of brushing and interdental cleaning	0.65	-0.14	-
Parents should help their kids (up to 6 years old) to brush properly	0.59	-0.53	-
Expected social outcomes[b]			
People judge each other on the basis of their teeth	0.42	0.89	-

I appreciate it when people with whom I socialize have well-maintained teeth	0.62	-0.41	-
In social contacts, oral health with fresh breath is important	0.66	-0.18	-
Benefits of brushing[b]			
Brushing my teeth at least two times a day will prevent tooth decay and gum disease	0.41	-0.39	-
If I brush my teeth at least two times a day they will last a lifetime	0.43	-0.44	-
My mouth feels better after I brush them	0.40	0.52	-
My breath feels better after I brush them	0.39	0.53	-
Brushing my teeth at least two times a day will save me money on dental expenses	0.42	-0.28	-
My teeth will look better if I brush at least two times a day	0.40	0.14	-
Barriers to brushing[b]			
Tooth brushing is painful	0.41	-0.50	0.16
My teeth will break when I brush	0.33	-0.40	0.13
My gums will bleed when I brush	0.36	0.33	-0.05
I forget to brush at least two times a day	0.37	0.27	-0.54
If I am tired I don't brush my teeth	0.45	0.25	-0.34
Toothpaste is expensive	0.07	0.28	-0.07
I don't have time to brush my teeth at least two times a day	0.39	0.19	0.06
I feel that my family didn't encourage me to brush my teeth regularly	0.21	0.32	0.52
I don't know how to brush my teeth properly	0.26	0.37	0.52
Self-efficacy with brushing[d]			
When you don't have time	0.56	-	-
When you are under a lot of stress	0.59	-	-
When you are anxious	0.58	-	-
Benefit of interdental cleaning[b]			
Cleaning the spaces between my teeth at least once a day will prevent tooth decay and gum disease	0.43	-	-
If I clean the spaces between my teeth at least once a day they will last a lifetime	0.41	-	-
My mouth feels better after I clean the spaces between my teeth	0.39	-	-
My breath is fresher after I clean the spaces between my teeth	0.41	-	-
Cleaning the spaces between my teeth at least once a day will save me money on dental expenses	0.44	-	-
My mouth will look better if I clean the spaces between my teeth once a day	0.38	-	-
Barriers to interdental cleaning[b]			
Interdental cleaning is painful	0.37	0.42	-0.34
My teeth will break when I clean between my teeth	0.42	0.20	-0.11
My gums will bleed when I clean between my teeth	0.36	0.37	-0.31
I forget to clean between my teeth at least once a day	0.31	-0.54	-0.26

If I am tired I don't clean between my teeth	0.33	-0.48	-0.12
Dental floss/tools for cleaning between my teeth is expensive	0.27	0.08	0.31
I don't have time to clean between my teeth at least once a day	0.37	-0.32	0.09
I feel that my family didn't encourage me to clean between my teeth regularly	0.22	0.06	0.58
I do not know how to clean the spaces between my teeth properly	0.31	0.10	0.51
Self-efficacy with interdental cleaning[e]			
When you don't have time	0.57	-	-
When you are under a lot of stress	0.59	-	-
When you are anxious	0.58	-	-

[a] Loadings represent the strength and direction of the association between each original variable and the extracted principal components. Higher absolute values indicate a stronger contribution of the variable to the corresponding component. The represented loadings are based on the penalized non-linear principal component analysis for each domain independently. Therefore, the number of principal component varies from domain to another. The number of principal components retained per domain was based on the proportion of explained variance (≥50%). Some domains yielded only one meaningful principal component due to high internal consistency among included variables. Knowledge domain was excluded from the principal component analysis because of the high polarity of the ordinal variables that hindered the excution of the analysis.

[b] To what extent do you agree (Strongly disagree; Disagree; Neutral; Agree; Strongly agree)

[c] Please select what applies to you: (Not serious ; A little serious ;Partially serious; Serious; Very serious)

[d] How confident are you that you will brush your teeth for 2 min twice daily on the circumstances below? (Not confident; A bit confident ;Fairly confident ; Quite confident ; Very confident)

[e] How confident are you that you will clean between your teeth once a day on the circumstances below? (Not confident; A bit confident ;Fairly confident ; Quite confident ; Very confident).

Supplementary Table S4

Table S4. Checklist of STROBE statement[a] items that are included in the current manuscript

Section	Item No	Recommendation	Included (Yes/No)?	If "Yes", where?	If "No", why?
**Title and abstract**	1	(*a*) Indicate the study's design with a commonly used term in the title or the abstract	Yes	Title and abstract	-
		(*b*) Provide in the abstract an informative and balanced summary of what was done and what was found	Yes	-	-
**Introduction**					
Background/rationale	2	Explain the scientific background and rationale for the investigation being reported	Yes	Line 72-79	-
Objectives	3	State specific objectives, including any prespecified hypotheses	Yes	Line 80	-
**Methods**					
Study design	4	Present key elements of study design early in the paper	Yes	Line 99	-
Setting	5	Describe the setting, locations, and relevant dates, including periods of recruitment, exposure, follow-up, and data collection	Yes	Line 100	-
Participants	6	(*a*) Give the eligibility criteria, and the sources and methods of selection of participants	Yes	Line 100	-
Variables	7	Clearly define all outcomes, exposures, predictors, potential confounders, and effect modifiers. Give diagnostic criteria, if applicable	Yes	Line 123	-
Data sources/ measurement	8*	For each variable of interest, give sources of data and details of methods of assessment (measurement). Describe comparability of assessment methods if there is more than one group	Yes	Lines 123-153 and questionnaire in supplementary file 1	-

Bias	9	Describe any efforts to address potential sources of bias	Yes	Across statistical analyses	-
Study size	10	Explain how the study size was arrived at	Yes	Line 103	-
Quantitative variables	11	Explain how quantitative variables were handled in the analyses. If applicable, describe which groupings were chosen and why	Yes	Lines 123-153	-
Statistical methods	12	(*a*) Describe all statistical methods, including those used to control for confounding	Yes	Lines 155-213	-
		(*b*) Describe any methods used to examine subgroups and interactions	-	-	-
		(*c*) Explain how missing data were addressed	Yes	Lines 229	-
		(*d*) If applicable, describe analytical methods taking account of sampling strategy	-	-	-
		(*e*) Describe any sensitivity analyses	Yes	Lines 205 and 226	-
**Results**					
Participants	13*	(a) Report numbers of individuals at each stage of study—eg numbers potentially eligible, examined for eligibility, confirmed eligible, included in the study, completing follow-up, and analysed	No	-	Since the majority of recruited participants were via snowballing, the eligibility was confirmed at an earlier stage.
		(b) Give reasons for non-participation at each stage	No	-	There was no cases of non-participation
		(c) Consider use of a flow diagram	No	-	A flow diagram was not included, as the number of recruited participants corresponded exactly to the predetermined sample size, with no exclusions or losses after recruitment.

Descriptive data	14*	(a) Give characteristics of study participants (eg demographic, clinical, social) and information on exposures and potential confounders	Yes	Lines 233	-
		(b) Indicate number of participants with missing data for each variable of interest	Yes	Lines 229	-
Outcome data	15*	Report numbers of outcome events or summary measures	Yes	Table 1	-
Main results	16	(*a*) Give unadjusted estimates and, if applicable, confounder-adjusted estimates and their precision (eg, 95% confidence interval). Make clear which confounders were adjusted for and why they were included	No	-	Due to the large amount of results and multi-phase analysis, the unadjusted estimates were included in the supplementary material.
		(*b*) Report category boundaries when continuous variables were categorized	-	-	-
		(*c*) If relevant, consider translating estimates of relative risk into absolute risk for a meaningful time period	-	-	-
Other analyses	17	Report other analyses done—eg analyses of subgroups and interactions, and sensitivity analyses	Yes	Lines 205 and 226	-
**Discussion**					
Key results	18	Summarise key results with reference to study objectives	Yes	Lines 365-379	-
Limitations	19	Discuss limitations of the study, taking into account sources of potential bias or imprecision. Discuss both direction and magnitude of any potential bias	Yes	Lines 445	-
Interpretation	20	Give a cautious overall interpretation of results considering objectives, limitations, multiplicity of analyses, results from similar studies, and other relevant evidence	Yes	Across all discussion	-

Generalisability	21	Discuss the generalisability (external validity) of the study results	Yes	Lines 454-457	-
**Other information**					
Funding	22	Give the source of funding and the role of the funders for the present study and, if applicable, for the original study on which the present article is based	No	-	It was mentioned during the submission process

[a] Source: www.strobe-statement.org

Supplementary Table S5

Table S5. Assessment of multicollinearity among predictors in the mixed-effects logistic regression model with adequate toothbrushing[a] as the outcome

**Explanatory Variable**		**df**	**GVIF**	**GVIF^(1/(2·df))**
	Sex	1	13.03	3.61
	Age	1	2.83	1.68
	Highest educational level	3	6.89	1.38
How much do you agree with the following[b]	I feel that I am in control of the decisions related to my dental health	1	1.75	1.32
How much do you agree with the following[b]	My mouth feels better after I brush my teeth	1	1.33	1.15
How much do you agree with the following[b]	Tooth brushing is painful	1	5.60	2.37
	I don’t have time to brush my teeth at least two times a day	1	4.29	2.07
	I forget to brush at least two times a day	1	3.28	1.81
How confident are you that you will brush your teeth for 2 min twice daily on the circumstances below[c]	When you don’t have time	1	7.83	2.80
	When you are under a lot of stress	1	6.90	2.63
	When you are anxious	1	2.65	1.63

[a] Adequate toothbrushing is toothbrushing at least twice a day versus less frequent.
[b] The answer is an agreement 5-point Likert scale (see Questionnaire in supplementary file 1).
[c] The answer is a confidence 5-point Likert scale (see Questionnaire in supplementary file 1).
Df= degree of freedom. GVIF = generalized variance inflation factor. GVIF was used instead of the standard Variance Inflation Factor because models were fitted using generalized linear mixed-effects models. For categorical variables with more than two levels, GVIF values were adjusted for degrees of freedom and reported as GVIF^(1/(2·df)). Fox, J. and Monette, G. (1992) Generalized collinearity diagnostics. JASA, 87, 178–183.

Supplementary Table S6

Table S6. Assessment of multicollinearity among predictors in the mixed-effects logistic regression model with adequate interdental cleaning[a] as the outcome

**Explanatory Variable**		**df**	**GVIF**	**GVIF^(1/(2·df))**
	Sex	1	1.09	1.04
	Age	1	1.41	1.19
	Highest educational level	3	1.44	1.06
How much do you agree with the following[b]	My mouth feels better after I clean the spaces between my teeth	1	1.69	1.30
	My breath is fresher after I clean the spaces between my teeth	1	1.85	1.36
	Cleaning the spaces between my teeth at least once a day will save me money on dental expenses	1	1.39	1.18
How much do you agree with the following[b]	I do not know how to clean the spaces between my teeth properly	1	1.16	1.08
	Dental floss/tools for cleaning between my teeth is expensive	1	1.15	1.07
	My teeth will break when I clean between my teeth	1	1.12	1.06

	I feel that my family didn't encourage me to clean between my teeth regularly	1	1.25	1.12
How confident are you that you will clean between your teeth once a day on the circumstances below[c]	When you are under a lot of stress	1	1.20	1.10

[a] Adequate interdental cleaning is cleaning at least every second day versus less frequent.

[b] The answer is an agreement 5-point Likert scale (see Questionnaire in supplementary file 1).

[c] The answer is a confidence 5-point Likert scale (see Questionnaire in supplementary file 1).

Df= degree of freedom. GVIF = generalized variance inflation factor. GVIF was used instead of the standard Variance Inflation Factor because models were fitted using generalized linear mixed-effects models. For categorical variables with more than two levels, GVIF values were adjusted for degrees of freedom and reported as GVIF^(1/(2·df)). Fox, J. and Monette, G. (1992) Generalized collinearity diagnostics. JASA, 87, 178–183.

Supplementary Figure S1

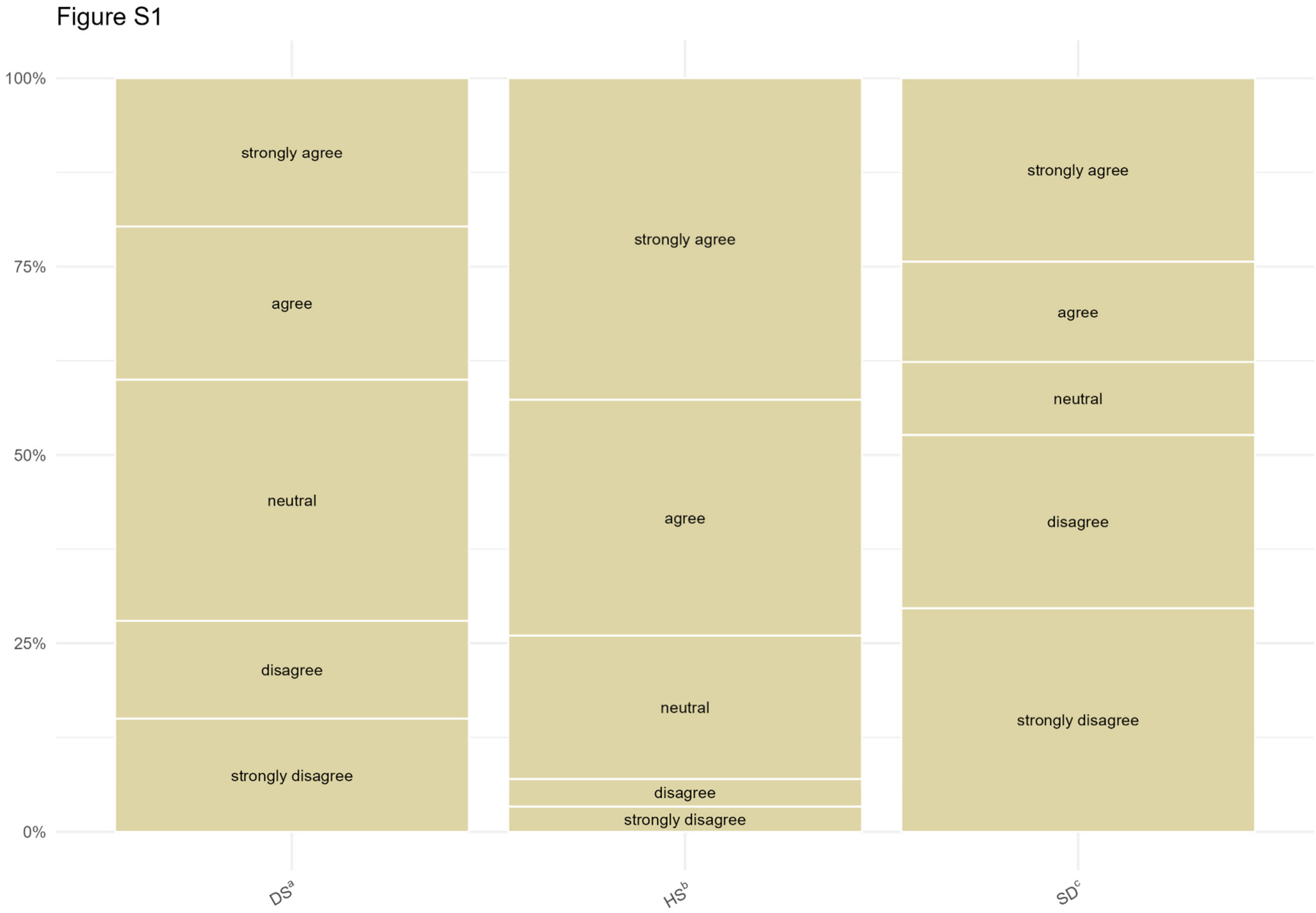

Figure S1. Response Distributions for Perception Domain's Variables.

([a–c]) To what extent do you agree with the following. DS[a]: Swiss dental care system cares about my well-being; HS[b]: Swiss healthcare system cares about my well-being; SD[c]: I feel that I am in control of decisions related to my dental health (autonomy). Access to oral health care among asylum seekers and refugees in Switzerland, Switzerland, 2025.

Supplementary Figure S2

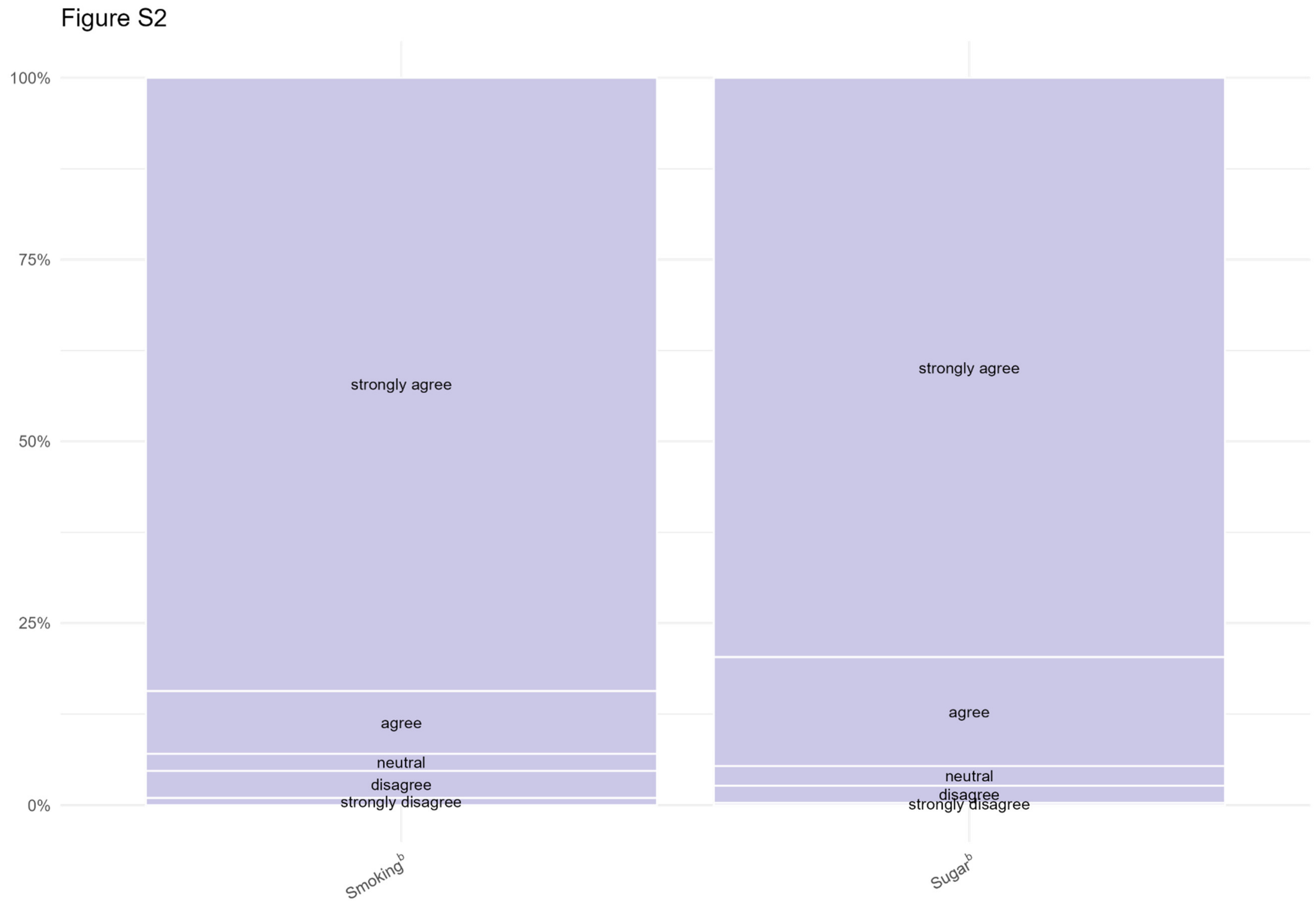

Figure S2. Response Distributions for Knowledge Domain's Variables. ([a]–[b])To what extent do you agree with the follwoing. Sugar[a]:Sugary food and drinks affect your teeth; Smoking[b]:Smoking can affect your oral health. Access to oral health care among asylum seekers and refugees in Switzerland, Switzerland, 2025.

Supplementary Figure S3

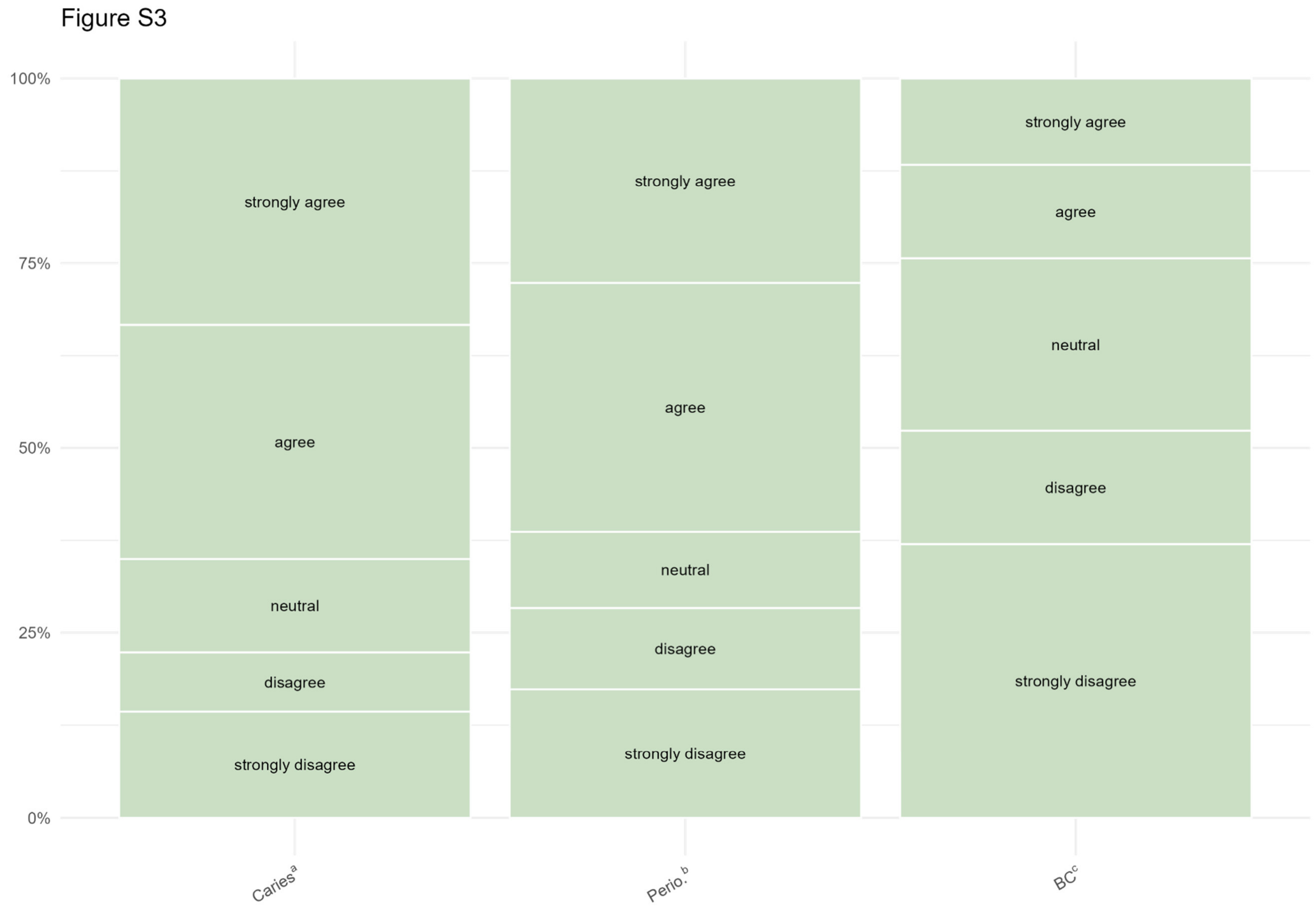

Figure S3. Response Distributions for Susceptibility Domain's Variables. ([a–c]) To what extent do you agree with the following? Caries[a]: There is a chance that I will get caries. Perio.[b]: There is a chance that I will have periodontal disease. BC[c]: My mouth is in bad condition.

Supplementary Figure S4

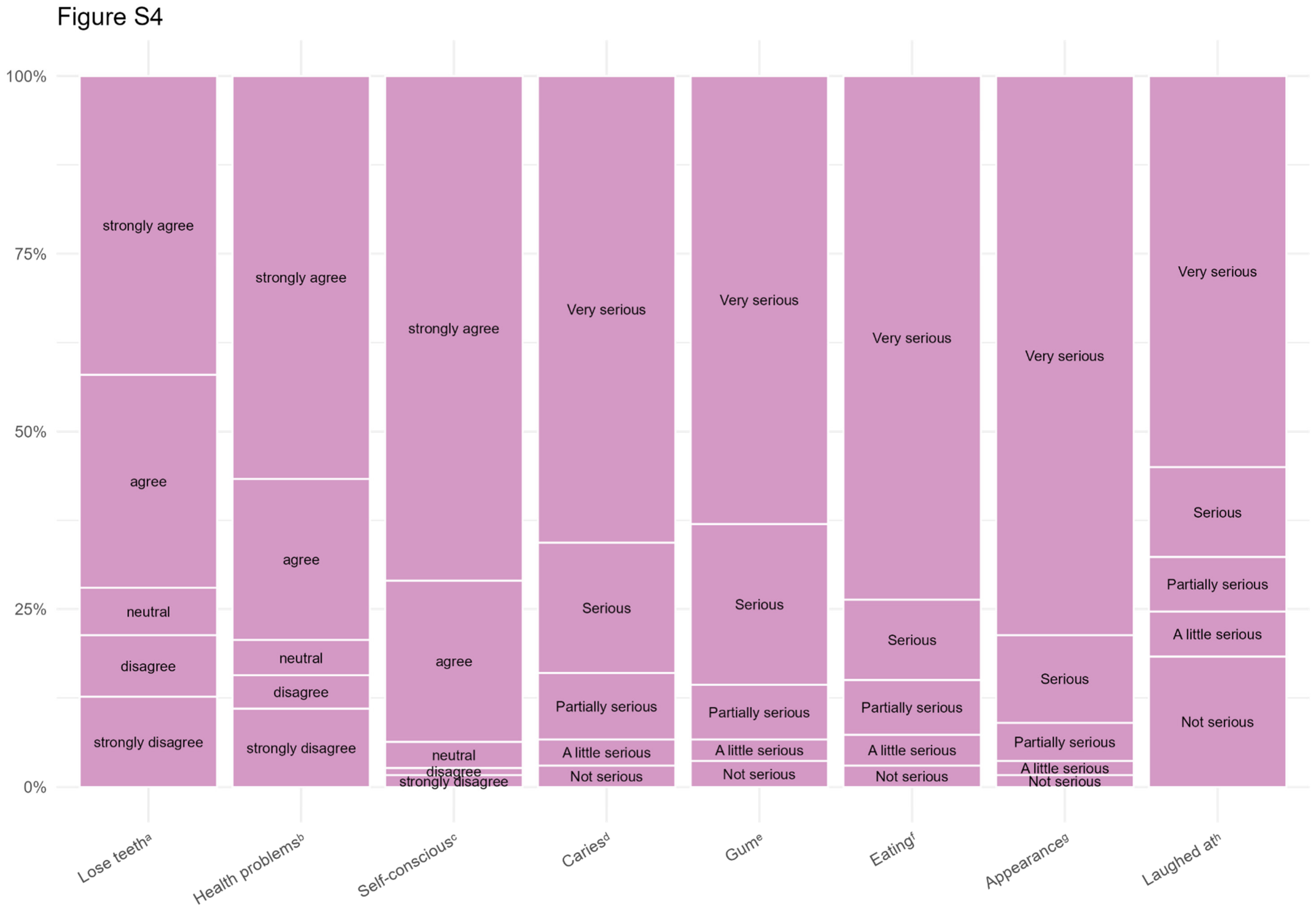

Figure S4. Response Distributions for Severity Domain's Variables. ([a]–[h]) Please select what applies to you: [a] I will lose my teeth if I get tooth decay or gum disease; [b] Tooth decay and gum diseases can cause other health problems; [c]My self-consciousness will be impaired if I have poor oral health conditions; [d] If I have caries, for me that is …; [e] If I have gum disease, for me that is …; [f] If I can't eat my favorite food because of oral diseases, for me that is …; [g] If my teeth do not look good because of oral diseases, for me that is …; [h] If I get laughed at by friends, relatives, or classmates because of oral diseases, for me that is …

Supplementary Figure S5

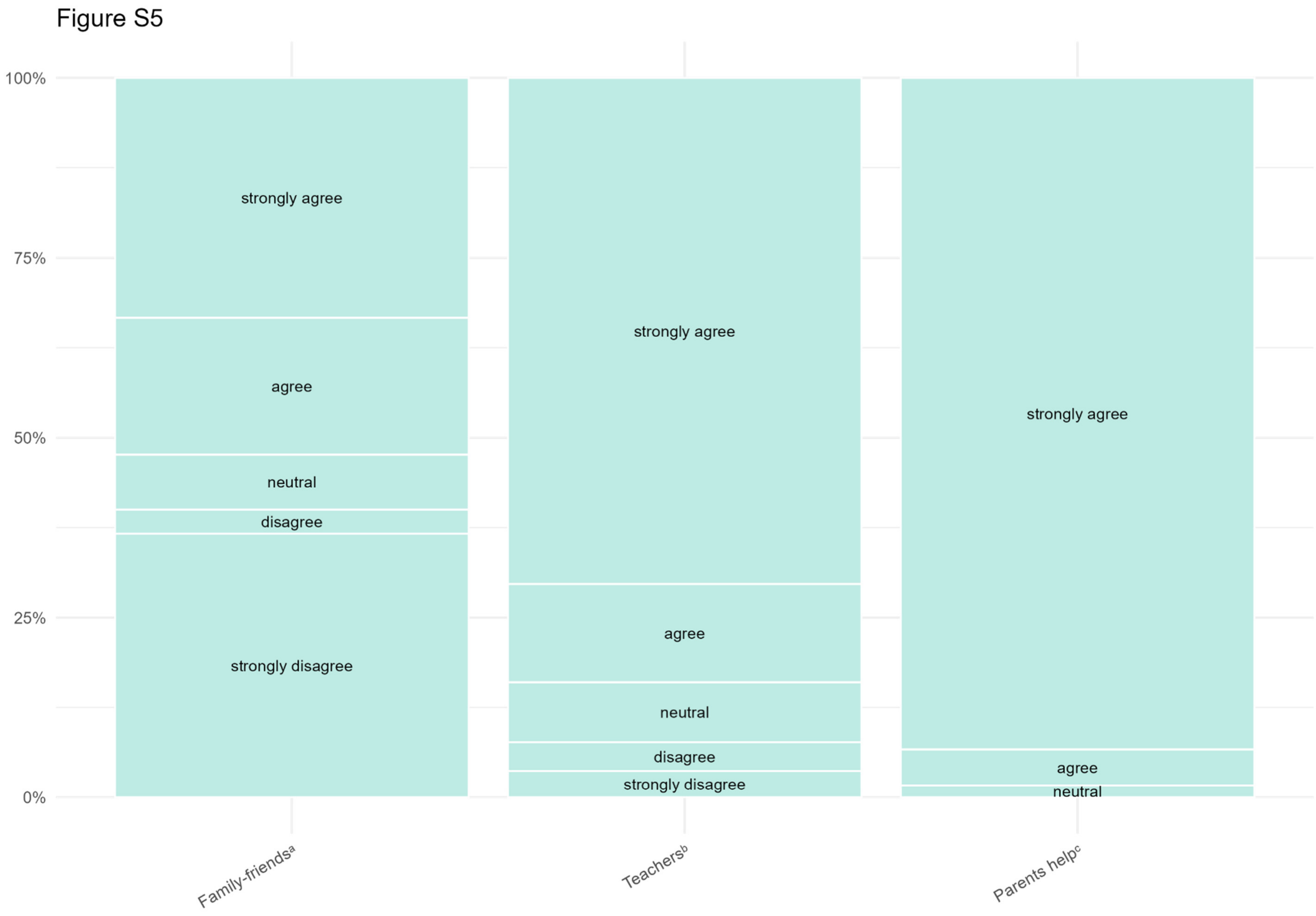

Figure S5. Response distributions for cues to action variables. ([a]–[c]) To what extent do you agree? [a] My parents often remind me of brushing and flossing (adolescents) / My family or friends often remind me of brushing and flossing (adults); [b] Teachers often remind their students of brushing and interdental cleaning; [c] Parents should help their children (up to 6 years old) to brush properly.

Supplementary Figure S6

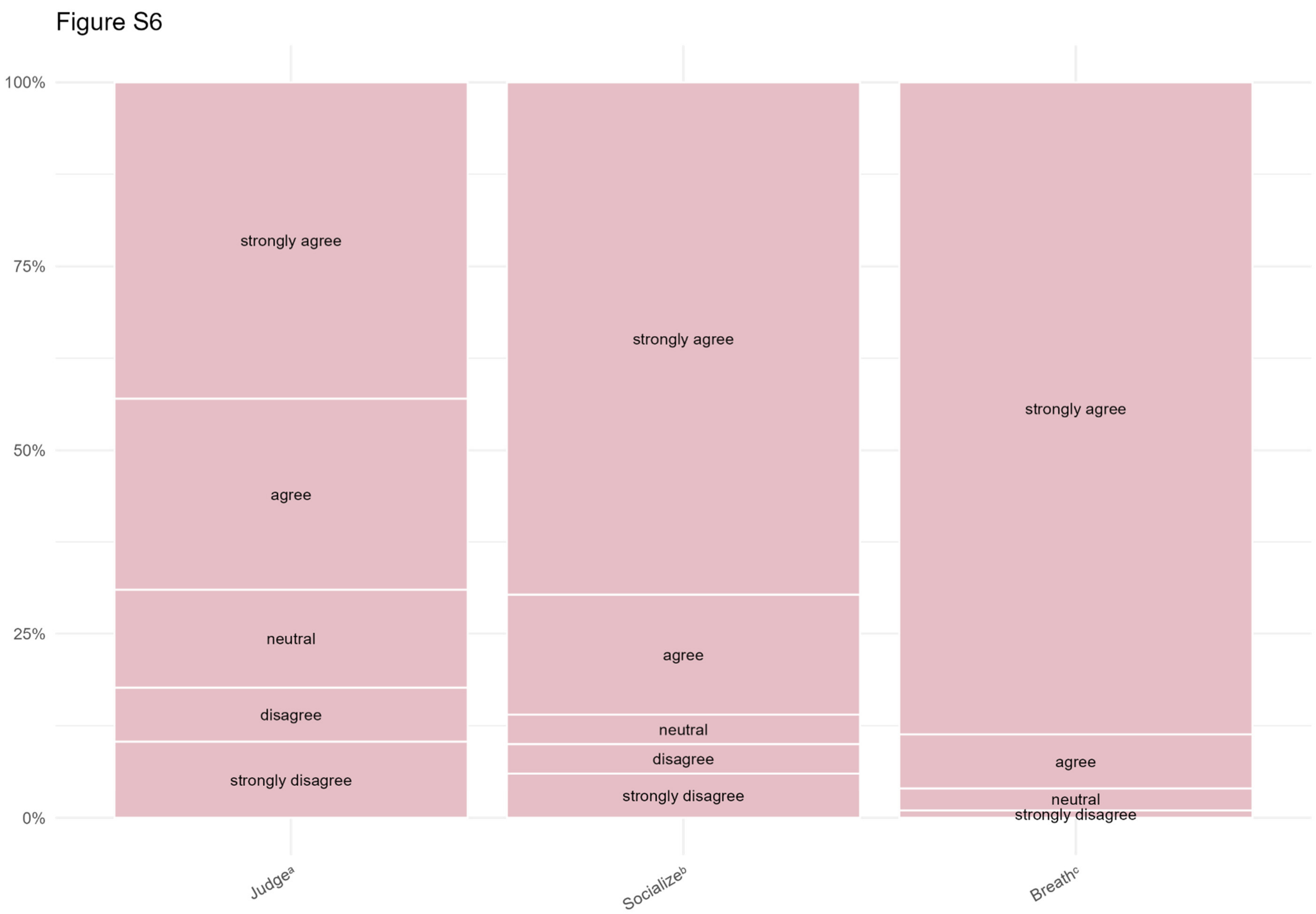

Figure S6: Response distributions for Expected social outcomes variables. ([a–c]) To what extent do you agree? [a]People judge each other on the basis of their teeth; [b]I appreciate it when people with whom I socialize have well maintained teeth; [c]In social contacts, oral health with fresh breath is important

Supplementary Figure S7

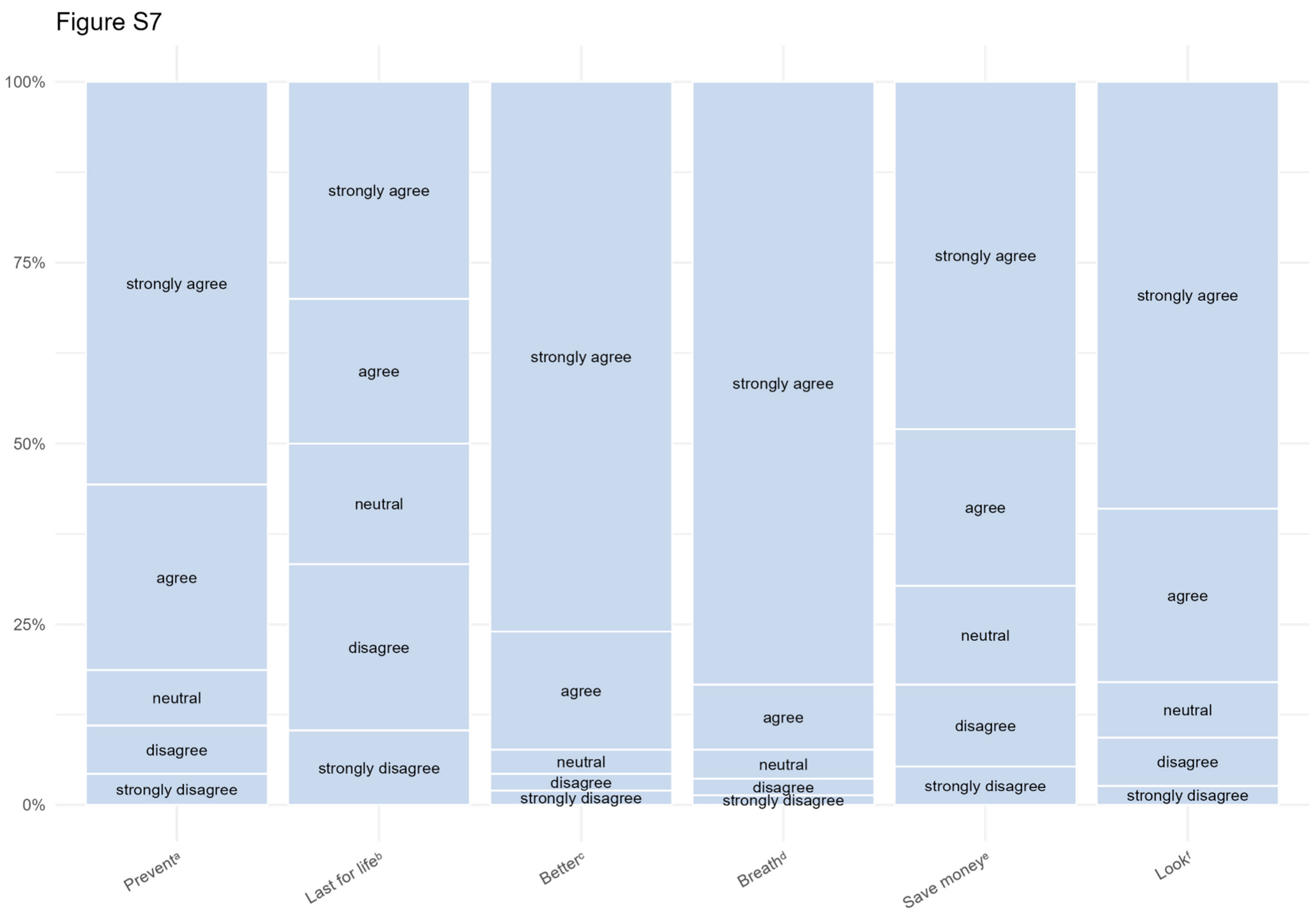

Figure S7: Response distributions for perceived benefits of toothbrushing twice a day. ([a–f]) To what extent do you agree with the following? \n[a] Brushing my teeth at least two times a day will prevent tooth decay and gum disease; [b] If I brush my teeth at least two times a day they will last a lifetime;\n[c] My mouth feels better after I brush my teeth; [d] My breath is fresher after I brush my teeth;\n[e] Brushing my teeth at least two times a day will save me money on dental expenses; [f] My teeth will look better if I brush at least two times a day.

Supplementary Figure S8

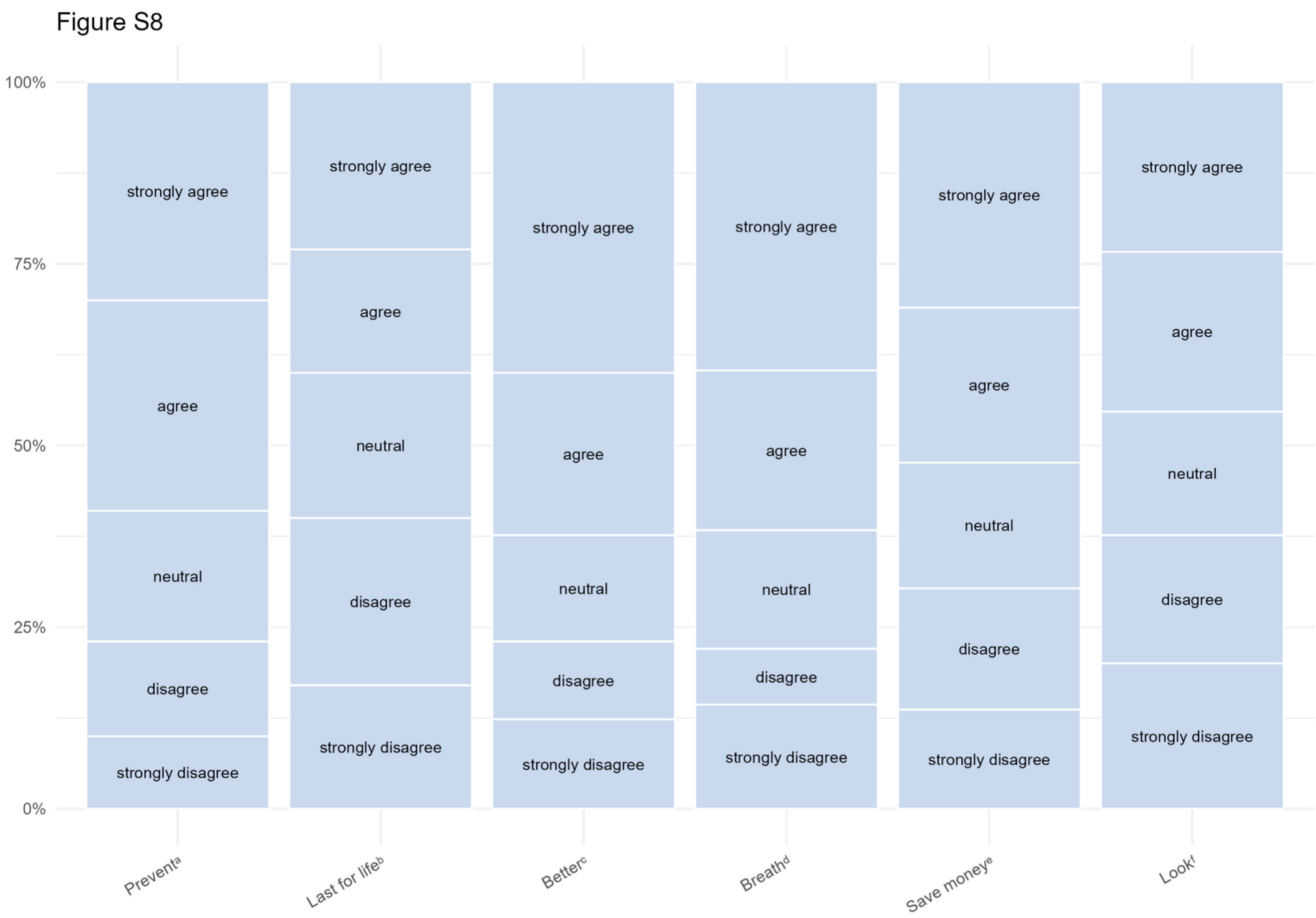

Figure S8. Response distributions for perceived benefits of interdental cleaning at least once a day. ([a]–[f]) To what extent do you agree with the following? [a] Cleaning the spaces between my teeth at least once a day will prevent tooth decay and gum disease; [b] If I clean the spaces between my teeth at least once a day they will last a lifetime; [c] My mouth feels better after I clean the spaces between my teeth; [d] My breath is fresher after I clean the spaces between my teeth; [e] Cleaning the spaces between my teeth at least once a day will save me money on dental expenses; [f] My mouth will look better if I clean the spaces between my teeth once a day.

Supplementary Figure S9

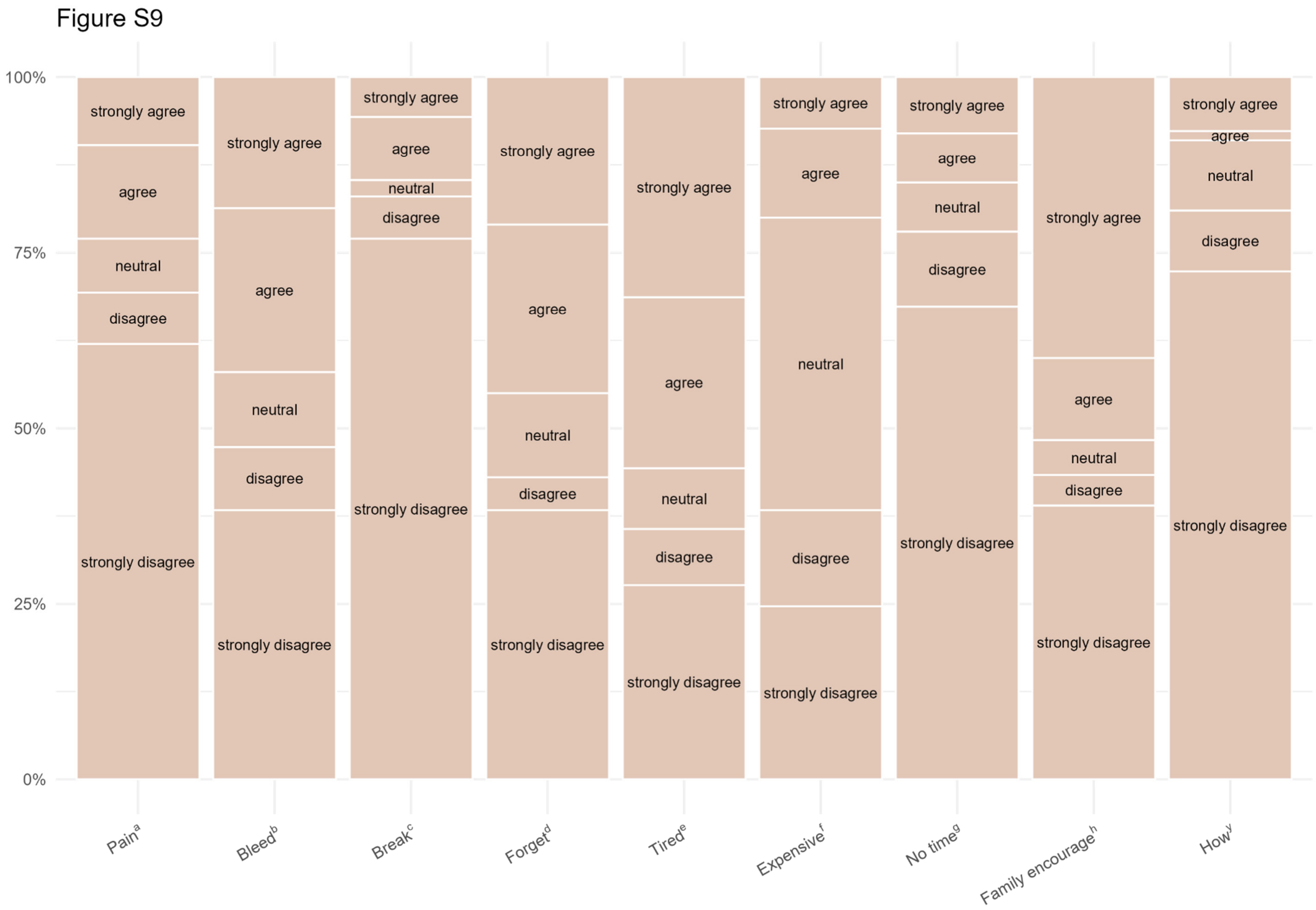

Figure S9: Response distributions for barriers to toothbrushing twice a day. ([a]–[y]) To what extent do you agree? [a] Tooth brushing is painful; [b] My gums will bleed when I brush; [c] My teeth will break when I brush; [d] I forget to brush at least two times a day; [e] If I am tired I don’t brush my teeth; [f] Toothpaste is expensive;\n[g] I don’t have time to brush my teeth at least two times a day; [h] I feel that my family didn’t encourage me to brush my teeth regularly; [y] I don’t know how to brush my teeth properly.

Supplementary Figure S10

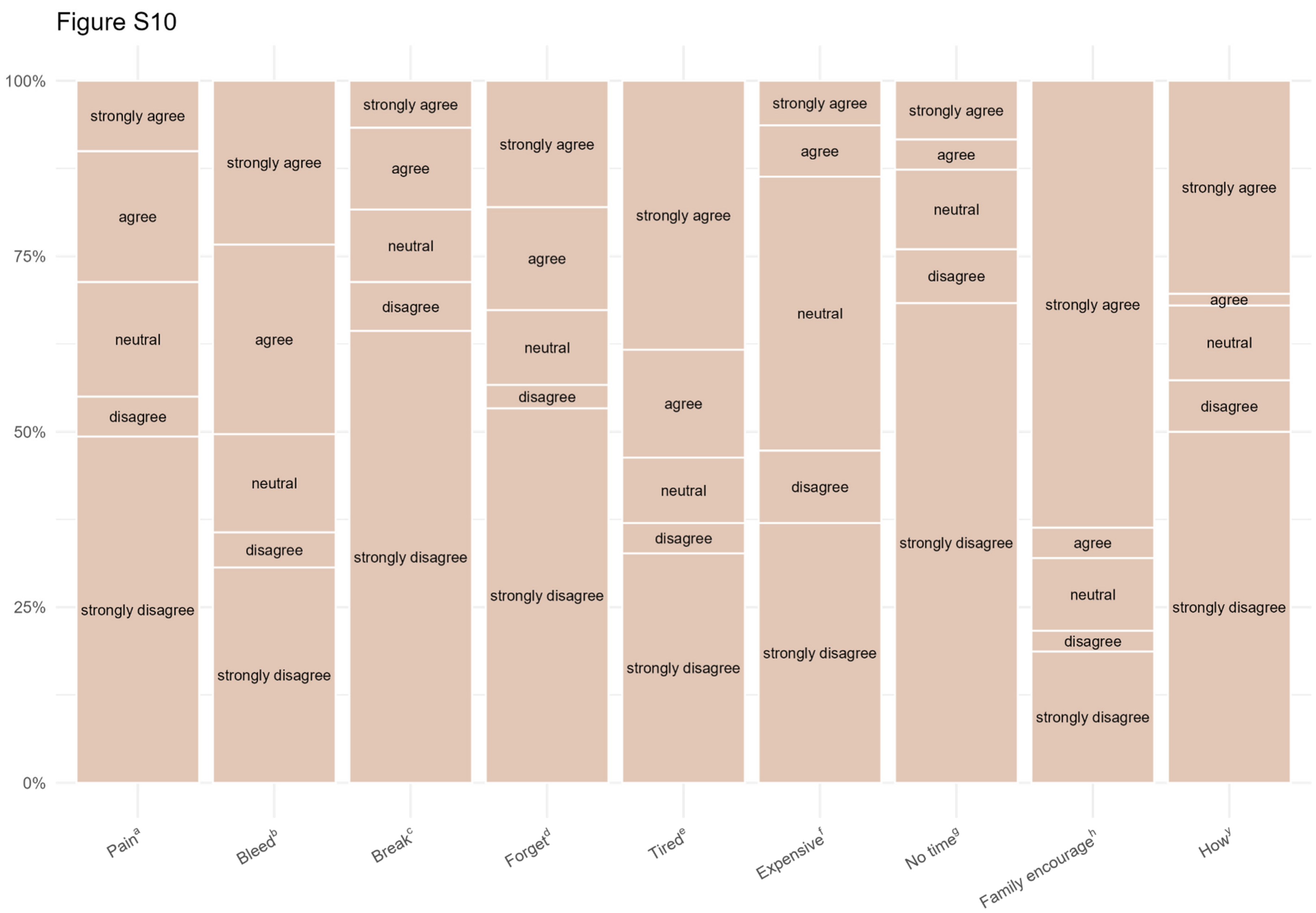

Figure S10. Response distributions for barriers to interdental cleaning once a day. ([a]–[y]) To what extent do you agree? [a] Interdental cleaning is painful; [b] My gums will bleed when I clean between my teeth; [c] My teeth will break when I clean between my teeth; [d] I forget to clean between my teeth at least once a day; [e] If I am tired I don’t clean between my teeth; [f] Dental floss/tools for cleaning between my teeth are expensive; [g] I don’t have time to clean between my teeth at least once a day; [h] I feel that my family didn’t encourage me to clean between my teeth regularly; \n[y] I do not know how to clean the spaces between my teeth properly.

Supplementary Figure S11

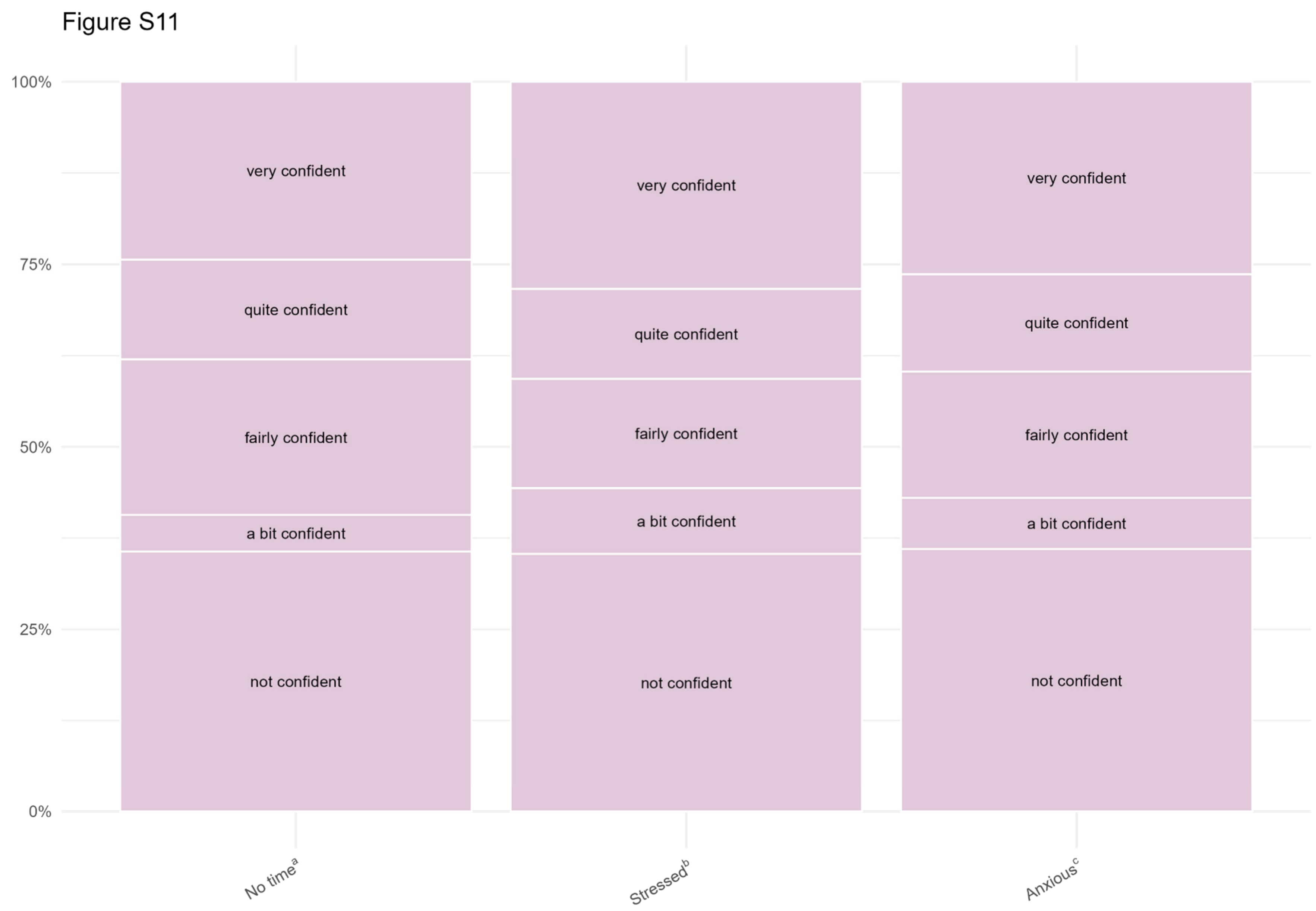

Figure S11: Response distributions for self-efficacy in toothbrushing. ([a]–[c]) How confident are you that you will brush your teeth for 2 minutes twice daily in the following situations? [a] When you don't have time; [b] When you are under a lot of stress; [c] When you are anxious.

Supplementary Figure S12

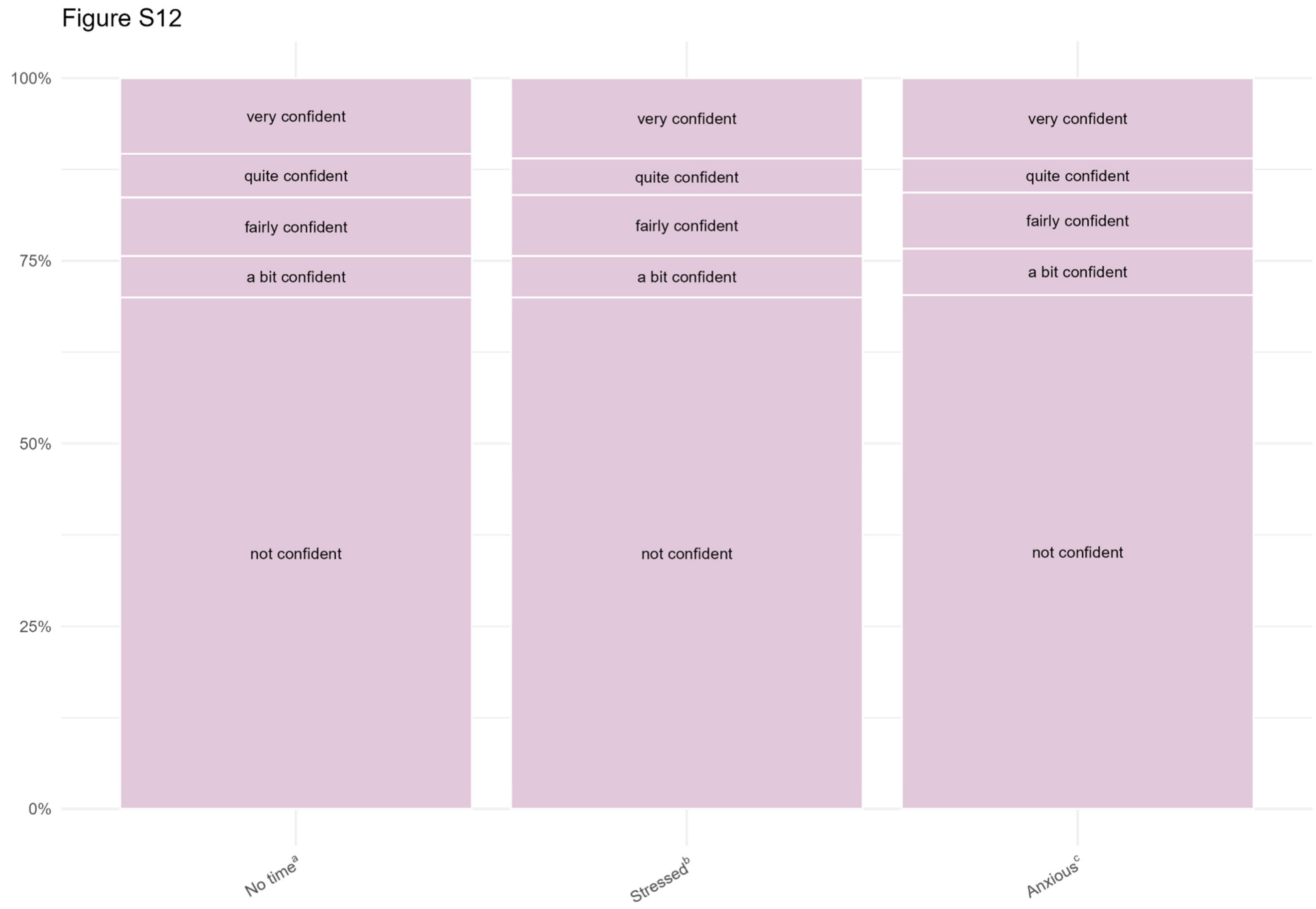

Figure S12: Response distributions for self-efficacy in interdental cleaning. ([a]–[c]) How confident are you that you will clean between your teeth once a day in the following situations? [a] When you don’t have time; [b] When you are under a lot of stress; [c] When you are anxious.

Supplementary Figure S13

Figure S13

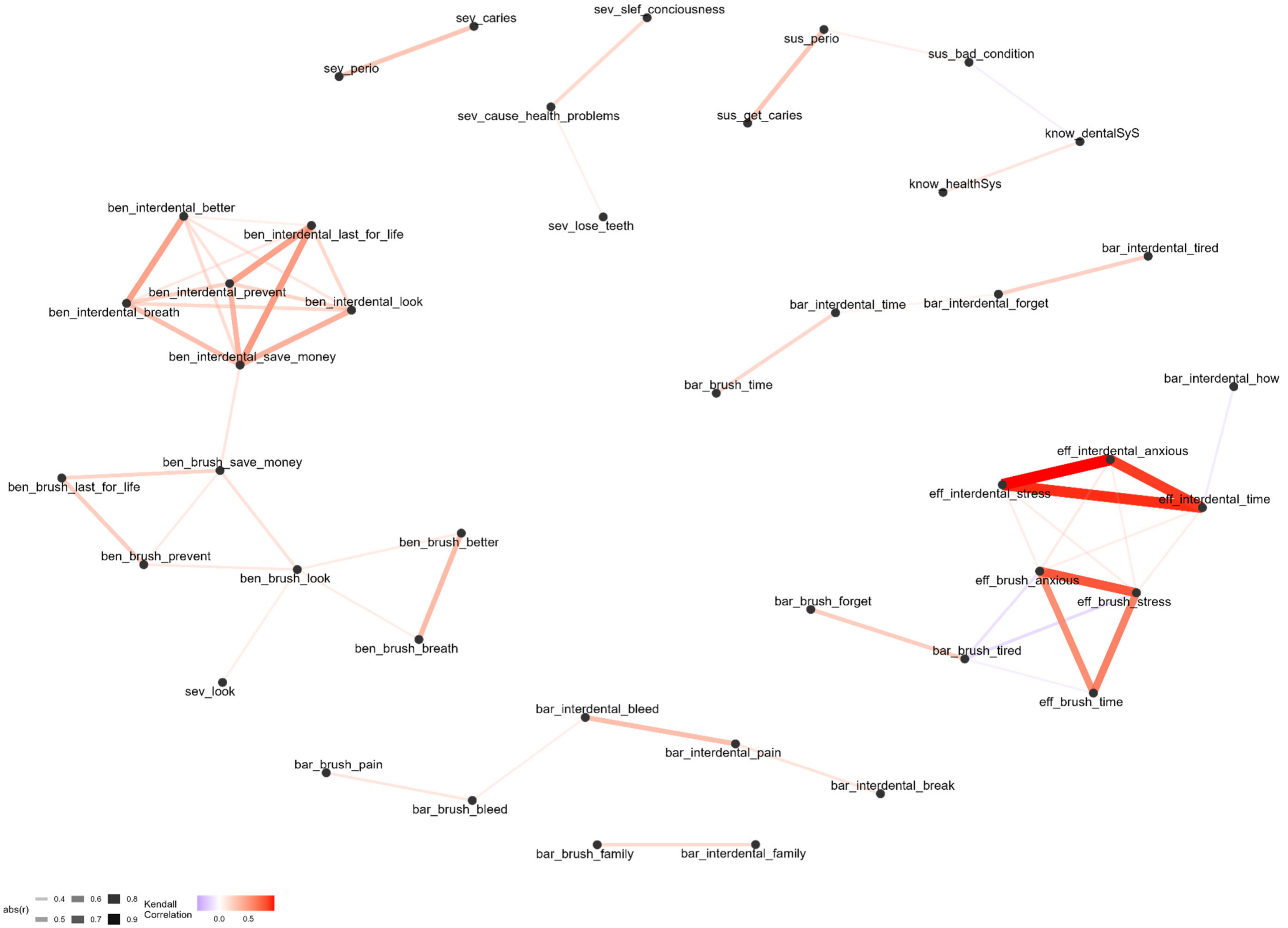

Figure S13 :Kendall's Tau ordinal association between original variables (|τ| > 0.3). Blue edges indicate negative correlations, red edges positive correlations. Edge width and opacity reflect the strength (|τ|) of the association. Only associations that are > 0.3, are displayed in the figure. To what extend do you agree (Strongly disagree; Disagree; Neutral; Agree; Strongly agree): know_dentalSyS (Perception):Swiss dental care system care about my well-being , know_healthSys (Perception):Swiss healthcare system cares about my well-being, sus_get_caries (Susceptibility):There is a chance that I will get caries, sus_perio (Susceptibility): There is a chance that I will have periodontal disease, sus_bad_condition (Susceptibility):My mouth is in bad condition, sev_lose_teeth (Severity): I will lose my teeth if I get tooth decay or gum disease, sev_cause_health_problems (Severity):Tooth decay and gum diseases can cause other health problems, sev_self_consciousness (Severity):My self-consciousness will be impaired if I have poor oral health conditions. Select what applies (Not serious ; A little serious ;Partially serious; Serious; Very serious): sev_caries (Severity): If I have caries, for me that is. To what extend do you agree (Strongly disagree; Disagree; Neutral; Agree; Strongly agree): ben_brush_look (Benefits of toothbrushing): My teeth will look better if I brush at least two times a day, ben_brush_prevent (Benefits of brushing): Brushing my teeth at least two times a day will prevent tooth decay and gum disease , ben_brush_last_for_life (Benefits of brushing): If I brush my teeth at least two times a day they will last a lifetime, ben_brush_better (Benefits of brushing): My mouth feels better after I brush them, ben_brush_breath (Benefits of brushing): My breath is fresher after I brush them, ben_brush_save_money (Benefits of brushing): Brushing my teeth at least two times a day will save me money on dental expenses, ben_interdental_look (Benefits of interdental cleaning): My mouth will look better if I clean the spaces between my teeth once a day, ben_interdental_prevent (Benefits of interdental cleaning): Cleaning the spaces between my teeth at least once a day will prevent tooth decay and gum disease, ben_interdental_last_for_life (Benefits of interdental cleaning): If I clean the spaces between my teeth at least once a day they will last a lifetime, ben_interdental_better (Benefits of interdental cleaning): My mouth feels better after I clean the spaces between my teeth, ben_interdental_breath (Benefits of interdental cleaning): My breath is fresher after I clean the spaces between my teeth, ben_interdental_save_money (Benefits of interdental cleaning): Cleaning the spaces between my teeth at least once a day will save me money on dental expenses, bar_brush_pain (Barriers to brushing): Tooth brushing is painful, bar_brush_bleed (Barriers to brushing): My gums will bleed when I brush, bar_brush_forget (Barriers to brushing): I forget to brush at least two times a day, bar_brush_tired (Barriers to brushing): If I am tired I don't brush my teeth, bar_brush_time (Barriers to brushing): I don't have time to brush my teeth at least two times a day, bar_interdental_pain (Barriers to interdental cleaning): Interdental cleaning is painful, bar_interdental_bleed (Barriers to interdental cleaning): My gums will bleed when I clean between my teeth, bar_interdental_forget (Barriers to interdental cleaning): I forget to clean between my teeth at least once a day, bar_interdental_tired (Barriers to interdental cleaning): If I am tired I don't clean between my teeth, bar_interdental_time (Barriers to interdental cleaning): I don't have time to clean between my teeth at least once a day. How confident are you that you will brush your teeth for 2 min twice daily on the circumstances below? (Not confident; A bit confident ;Fairly confident ; Quite confident ; Very confident) (self-effiacy in brushing). eff_brush_time: When you don't have time, eff_brush_stress: When you are under a lot of stress, eff_brush_anxious: When you are anxious. How confident are you that you will clean between your teeth once a day on the circumstances below? (Not confident; A bit confident ;Fairly confident ; Quite confident ; Very confident) (Self-efficacy in interdental cleaning). eff_interdental_time: When you don't have time, eff_interdental_stress: When you are under a lot of stress, eff_interdental_anxious: When you are anxious.

Supplementary Questionnaire

Covariates

Age (years):-----

Sex (Male, Female, Other)

Country of birth:-----

Current residency permit:

N-permit (Asylum case under process)

F-permit (Provisionally admitted person)

F-permit (Provisionally admitted refugee)

B-permit (recognized refugee)

Highest educational level achieved: (Primary school, Secondary school, middle school or apprenticeship, Technical College or University, other:-------)

Year of arrival to Switzerland:-----

Determinants of oral hygiene behaviors:

Perceptions

Perceptions of dental and healthcare

To what extent do you agree: (Strongly disagree; Disagree; Neutral; Agree; Strongly agree)

Swiss **healthcare** system cares about my well-being

Swiss **dental care** system care about my well-being

Autonomy

To what extent do you agree: (Strongly disagree; Disagree; Neutral; Agree; Strongly agree)

I feel that I am in control of the decisions related to my dental health

Knowledge

To what extent do you agree: (Strongly disagree; Disagree; Neutral; Agree; Strongly agree)

Smoking can affect your oral health

Sugary food and drinks affect your teeth

Perceived susceptibility of oral disease

To what extend do you agree: (Strongly disagree; Disagree; Neutral; Agree; Strongly agree)

There is a chance that I will get caries.

There is a chance that I will have periodontal disease. (hint: Periodontal diseases are infections of the gums and bone surrounding the teeth, caused by bacteria in plaque buildup. Without treatment, these infections can lead to gum inflammation (gingivitis) and, if severe, can result in tooth and bone loss)

My mouth is in bad condition.

Perceived severity of oral disease

Please select what applies to you: (Not serious ; A little serious ;Partially serious; Serious; Very serious)

If I have caries, for me that is …

If I have gum disease, for me that is …

If my teeth do not look good because of oral diseases, for me that is …

If I can't eat my favorite food because of oral diseases, for me that is …

If I get laughed at by friends or relatives classmates because of oral diseases, for me that is

To what extend do you agree: (Strongly disagree; Disagree; Neutral; Agree; Strongly agree)

I will lose my teeth if I get tooth decay or gum disease

Tooth decay and gum diseases can cause other health problems

My self-consciousness will be impaired if I have poor oral health conditions.

Cues to action

To what extend do you agree: (Strongly disagree; Disagree; Neutral; Agree; Strongly agree)

My parents often remind me of brushing and flossing (Adolescents only)

My family or friends often remind me of brushing and flossing. (Adults only)

Teachers often reminds their students of brushing and interdental cleaning

Parent should help their kids (up to 6 years old) to brush properly

Expected social outcomes

To what extend do you agree: (Strongly disagree; Disagree; Neutral; Agree; Strongly agree)

People judge each other on the basis of their teeth

I appreciate it when people with whom I socialize have well maintained teeth

In social contacts, oral health with fresh breath is important

Benefits of brushing

To what extent do you agree with the following: (Strongly disagree; Disagree; Neutral; Agree; Strongly agree)

Brushing my teeth at least two times a day will prevent tooth decay and gum disease

If I brush my teeth at least two times a day they will last a lifetime

My mouth feels better after I brush them

My breath is fresher after I brush them

Brushing my teeth at least two times a day will save me money on dental expenses

My teeth will look better if I brush at least two times a day

Benefits of interdental cleaning

Hint to the participants: Interdental cleaning involves removing plaque and food particles from between the teeth where a regular toothbrush can't reach using dental floss and/or interdental brushes/sticks.

To what extent do you agree with the following: (Strongly disagree; Disagree; Neutral; Agree; Strongly agree)

Cleaning the spaces between my teeth at least once a day will prevent tooth decay and gum disease

If I clean the spaces between my teeth at least once a day they will last a lifetime

My mouth feels better after I clean the spaces between my teeth

My breath is fresher after I clean the spaces between my teeth

Cleaning the spaces between my teeth at least once a day will save me money on dental expenses

My mouth will look better if I clean the spaces between my teeth once a day

Barriers to brushing

To what extent do you agree: (Strongly disagree; Disagree; Neutral; Agree; Strongly agree)

Tooth brushing is painful

My teeth will break when I brush

My gums will bleed when I brush

I forget to brush at least two times a day

If I am tired I don’t brush my teeth

Toothpaste is expensive

I don’t have time to brush my teeth at least two times a day

I feel that my family didn’t encourage me to brush my teeth regularly

I don’t know how to brush my teeth properly

Barriers to interdental cleaning

To what extend do you agree: (Strongly disagree; Disagree; Neutral; Agree; Strongly agree)

Interdental cleaning is painful

My teeth will break when I clean between my teeth

My gums will bleed when I clean between my teeth

I forget to clean between my teeth at least once a day

If I am tired I don't clean between my teeth

Dental floss/tools for cleaning between my teeth is expensive

I don't have time to clean between my teeth at least once a day

I feel that my family didn't encourage me to clean between my teeth regularly

I do not know how to clean the spaces between my teeth properly

Self-efficacy in bushing

How confident are you that you will brush your teeth for **2 min twice daily** on the circumstances below?

(Not confident; A bit confident ;Fairly confident ; Quite confident ; Very confident)

When you don't have time

When you are under a lot of stress

When you are anxious

Self-efficacy in interdental cleaning

How confident are you that you will clean between your teeth **once a day** on the circumstances below? (Not confident; A bit confident ;Fairly confident ; Quite confident ; Very confident)

When you don't have time

When you are under a lot of stress

When you are anxious

Oral hygiene behavior:

Brushing

During the last week, how often did you brush your teeth? (not at all, once a week , every second day, once a day, twice a day)

Interdental cleaning

During the last week, how often did you clean the spaces between your teeth?

(not at all, once a week, every second day, once a day, twice a day)
